# Genetic Upregulation of Activated Protein C Mitigates Delayed Effects of Acute Radiation Exposure in the Mouse Plasma

**DOI:** 10.3390/metabo14050245

**Published:** 2024-04-24

**Authors:** Shivani Bansal, Yaoxiang Li, Sunil Bansal, William Klotzbier, Baldev Singh, Meth Jayatilake, Vijayalakshmi Sridharan, José A. Fernández, John H. Griffin, Hartmut Weiler, Marjan Boerma, Amrita K. Cheema

**Affiliations:** 1 Department of Oncology, Lombardi Comprehensive Cancer Center, Georgetown University Medical Center, Washington, DC 20057, USA; sm3451@georgetown.edu (S.B.); sb1886@georgetown.edu (S.B.); wek11@georgetown.edu (W.K.); bs1126@georgetown.edu (B.S.); mmj61@georgetown.edu (M.J.); 2 Division of Radiation Health, Department of Pharmaceutical Sciences, University of Arkansas for Medical Sciences, Little Rock, AR 72205, USA; vmohanseenivasan@uams.edu (V.S.); mboerma@uams.edu (M.B.); 3 Department of Molecular Medicine, Scripps Research Institute, La Jolla, CA 92037, USA; jfernand@scripps.edu (J.A.F.); jgriffin@scripps.edu (J.H.G.); 4 Versiti Blood Research Institute, Medical College of Wisconsin, Milwaukee, WI 53233, USA; hartmut.weiler@bcw.edu; 5 Departments of Biochemistry, Molecular and Cellular Biology, Georgetown University Medical Center, Washington, DC 20057, USA

**Keywords:** activated protein C, metabolomics, lipidomics, DEARE, radiation injury, C57BL/6N mouse

## Abstract

Exposure to ionizing radiation, accidental or intentional, may lead to delayed effects of acute radiation exposure (DEARE) that manifest as injury to organ systems, including the kidney, heart, and brain. This study examines the role of activated protein C (APC), a known mitigator of radiation-induced early toxicity, in long-term plasma metabolite and lipid panels that may be associated with DEARE in APCHi mice. The APCHi mouse model used in the study was developed in a C57BL/6N background, expressing the D168F/N173K mouse analog of the hyper-activatable human D167F/D172K protein C variant. This modification enables increased circulating APC levels throughout the mouse’s lifetime. Male and female cohorts of C57BL/6N wild-type and APCHi transgenic mice were exposed to 9.5 Gy γ-rays with their hind legs shielded to allow long-term survival that is necessary to monitor DEARE, and plasma was collected at 6 months for LC-MS-based metabolomics and lipidomics. We observed significant dyslipidemia, indicative of inflammatory phenotype, upon radiation exposure. Additionally, observance of several other metabolic dysregulations was suggestive of gut damage, perturbations in TriCarboxylic Acid (TCA) and urea cycles, and arginine metabolism. We also observed gender- and genotype-modulated metabolic perturbations post radiation exposure. The APCHi mice showed near-normal abundance for several lipids. Moreover, restoration of plasma levels of some metabolites, including amino acids, citric acid, and hypoxanthine, in APCHi mice is indicative of APC-mediated protection from radiation injuries. With the help of these findings, the role of APC in plasma molecular events after acute γ-radiation exposure in a gender-specific manner can be established for the first time.

## 1. Introduction

Exposure to ionizing radiation (IR) causes molecular and cellular damage, depending on the radiation type, dose, dose rate, and the genetic makeup of the exposed individual. Exposure to a single large dose of IR results in acute radiation syndrome (ARS), which could subsequently result in cumulative delayed effects of acute radiation exposure (DEARE) as time passes. ARS could manifest as gastrointestinal tract (GIT) injuries and hemopoietic disorders, while the survivors of ARS might experience delayed effects which include a myriad of chronic illnesses affecting multiple organ systems, including the skin, kidney, heart, and brain [1].

The general population is at risk of exposure to IR, as radiological events are largely unpredictable. Thus, radiation mitigators are needed to counteract both acute and delayed radiation toxicity, especially for emergency first responders or soldiers. Countermeasures that have been approved thus far are primarily aimed at alleviating acute radiation toxicities by enhancing bone marrow function. Currently, there are no FDA-approved therapeutics tailored against late radiation effects. Radiation mitigators have been found to improve endothelial cell (EC) function and reduce late organ injury, thus enhancing survival [2,3]. Endothelial and vascular dysfunction are thought to play a key role in radiation-induced multiple organ injury. Post radiation exposure, a loss of thrombin–thrombomodulin complex from EC leads to decreased activated protein C (APC) production and/or activation [4]. APC is a component of plasma that possesses potent anti-inflammatory, anti-coagulant, and cytoprotective properties [5]. The attenuation of IR-induced changes in the rat urine metabolome by two bolus injections of APC at 24 and 48 h following 13.0 Gy of partial body X-irradiation has been previously observed [6].

This study was designed to investigate the potential attenuation of delayed radiation effects in genetically altered mice expressing supraphysiologic levels of APC (APCHi) since the role of APC in the development of DEARE is still largely unexplored. The APCHi mouse model was generated on a C57BL/6N background that expresses the D168F/N173K mouse analog of the hyper-activatable human D167F/D172K protein C variant [7,8], which allows for increased circulating APC levels throughout the lifetime of the mouse. We have previously shown gender-dependent modulation in DEARE in the GIT, heart, and skin in APCHi mice [9].

Metabolomics is a tool of choice to delineate radiation-induced metabolic alterations and help identify potential differences based on gender or individual genotypes, as metabolomics can provide phenotypic signatures that are downstream of other -omics technologies [10]. Employing metabolomics to analyze and quantify changes in metabolic profiles can help to characterize the underlying physiological conditions of an individual well before symptoms of tissue injury or organ dysfunction become apparent [11]. In this study, male and female cohorts of C57BL/6N wild-type (WT) and APCHi transgenic mice were exposed to 9.5 Gy of γ-radiation to determine changes in plasma metabolic profiles. The goal of this study was two-fold: first, to identify the long-term metabolomic biomarkers of radiation injury at six months post-irradiation, and second, to understand how genotype and gender modulate delayed radiation response. We found that radiation exposure induced perturbations in mouse plasma profiles that were indicative of dyslipidemia and disruption of the metabolic alterations related to initial gut injuries, amino acid metabolism, and energy metabolism. Some of the perturbations were different between male and female mice and between the two genotypes, where APCHi mice showed fewer late effects of IR exposure.

## 2. Materials and Methods

### 2.1. Animal Procedures

All animal work was performed at the University of Arkansas for Medical Sciences (UAMS) under an approved protocol (#3763). The animal protocol approval date was 21 November 2019. A detailed description of the methods has been provided in a previous publication [9]. Briefly, we used male and female mice on a C57BL/6N background. Male and female mice carrying the D168F/N173K transgene (obtained from Hartmut Weiler, PhD, Blood Center of Wisconsin) were bred with WT C57BL/6N mice (Jackson Laboratories, Bar Harbor, ME, USA) to obtain APCHi and WT mice. Male and female WT and APCHi mice were randomly divided into sham and irradiated cohorts, as follows. Group 1 consisted of C57BL/6N wild type mice (*n* = 33). Of these 33 mice, 20 were males and 13 were females. Of these mice, 8 males and 8 females were assigned to sham treatment, and 12 male and 5 female mice received radiation treatment (9.5 Gy). Group 2 consisted of transgenic APCHi mice (*n* = 34). Of these 34 mice, 17 were males and 17 were females. There were 8 males and 9 females in the sham condition in group 2, while the radiation cohort (9.5 Gy) contained 9 males and 8 females.

At the age of 12–14 weeks, APCHi and WT mice of both genders (34 mice in total) were exposed to 9.5 Gy of γ-rays delivered at 1 Gy/min, using a 137Cs source cabinet irradiator (Mark 1, Model 68A, JL Shepherd & Associates, San Fernando, CA, USA) without the use of anesthesia. A separate cohort of mice was exposed to sham-irradiation by bringing them to the radiation room and placing them in the holders for 9.5 min without exposing them to radiation. Dosimetry was performed with Gafchromic film (DOSE-MAP, Ashland Specialty Ingredients, Wayne, NJ, USA) and an ion chamber (Exradin A20, Standard Imaging, Middleton, WI, USA) and electrometer (×4000, Standard Imaging) that are calibrated for γ-rays once a year. None of the animals reached these humane endpoints. All 67 animals survived until 6 months after irradiation. At 6 months after IR, animals were placed under anesthesia, and a blood sample was collected from the inferior vena cava into EDTA-coated tubes and immediately processed to prepare plasma. Plasma samples were stored at −80 °C until shipped on dry ice to Georgetown University Medical Center (Washington, DC, USA) for -omics analysis.

### 2.2. Chemicals

All LC-MS grade solvents, including acetonitrile and water, were purchased from Fisher Optima grade, Fisher Scientific. High-purity formic acid (99%) was purchased from Thermo-Scientific. Debrisoquine and 4-nitrobenzoic acid were purchased from Sigma-Aldrich (St. Louis, MO, USA). EquiSPLASH^®^ LIPIDOMIX^®^, 15:0-18:1-d7-PA, C15 Ceramide-d7 (d18:1-d7/15:0) and 18:1 Chol (D7) ester were purchased from Avanti Polar Lipids (Alabaster, AL, USA). Internal standards for free fatty acid (FFA), dihydroceramides (DCER), hexosylceramides (HCER), and lactosylceramides (LCER) were purchased from Sciex (Framingham, MA, USA) as Lipidyzer platform kit.

### 2.3. Untargeted Metabolomics Using UPLC-ESI-QTOF-MS

A total of 75 μL of water–methanol–isopropanol (3.5:2.5:4) containing internal standards (debrisoquine and 4-nitrobenzoic acid) was added to 25 µL of plasma sample. Next, the samples were vortexed and incubated at 4 °C for 20 min, followed by the addition of 100 μL of chilled acetonitrile to each sample and incubated at −20 °C for 20 min. Finally, the samples were centrifuged at 13,000 rpm for 20 min at 4 °C. The supernatant of each sample was transferred to MS vials for data acquisition. The sample queue was randomized to avoid bias. Each sample (1 μL) was injected into a 1.7 μm, 2.1 mm × 50 mm Acquity BEH C18 column (Waters Corporation, Milford, MA, USA) using an Acquity UPLC system connected to an electrospray ion source coupled with a quadrupole time-of-flight mass spectrometer (ESI-Q-TOF, Xevo-G2S, Waters Corporation, Milford, MA, USA) operating in positive and negative ionization modes. The data were acquired in centroid TOF-MS mode over a mass range from 50 to 1200 *m*/*z*.

### 2.4. Targeted Metabolomics and Lipidomics Using 5500 QTRAP

The targeted metabolomics and lipidomics methods were developed in-house to quantitate small endogenous molecules and lipids, respectively, using QTRAP^®^ 5500 LC-MS/MS System (Sciex, Framingham, MA, USA). An amount of 50 μL of chilled isopropanol containing internal standards was added to the 10 µL of each plasma sample. The samples were vortexed for 1 min and kept at 4 °C for 20 min, followed by incubation at −20 °C for 2 h. The samples were centrifuged at 13,000 rpm for 20 min at 4 °C. The supernatant was transferred to an MS vial for LC-MS analysis. An amount of 5 µL of the sample was injected onto a Kinetex 2.6 μm 100 Å 100 × 2.1 mm (Phenomenex, Torrance, CA, USA) for targeted metabolomics and Xbridge amide 3.5 µm, 4.6 × 100 mm (Waters, Milford, MA, USA) for targeted lipidomics using SIL-30 AC auto sampler (Shimazdu, Kytoto, Japan) connected with a high flow LC-30AD solvent delivery unit (Shimazdu, Kytoto, Japan) and CBM-20A communication bus module (Shimazdu, Kytoto, Japan) online with QTRAP 5500 (Sciex, Framingham, MA, USA) operating in positive and negative ion mode. The details on data processing and statistical analysis are provided in the Supplementary Materials.

## 3. Results

### 3.1. Radiation Exposure Elicits Robust Metabolic Response

**Figure 1:**
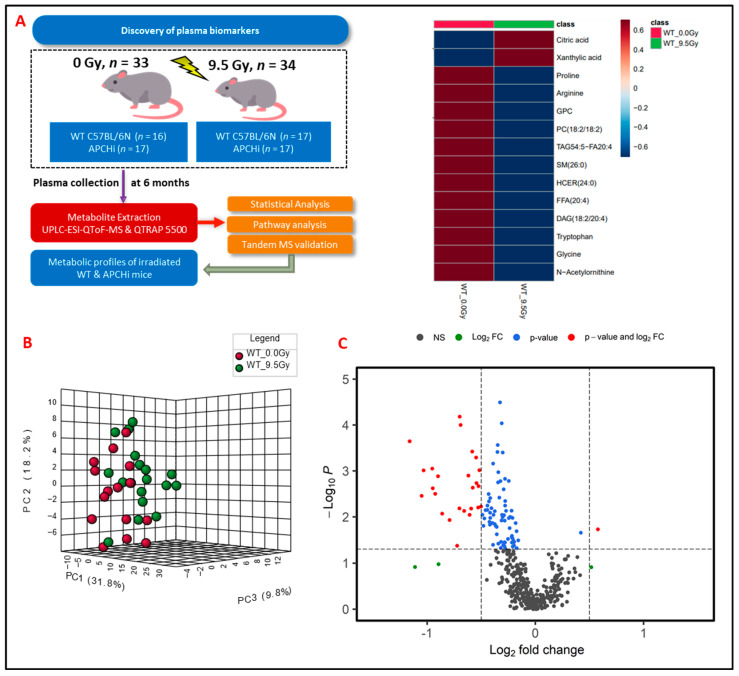
(**A**) Experimental and analytical design of the study. Six months post-treatment, plasma samples were gathered from both C57BL/6N and APCHi mice that underwent either irradiation (9.5 Gy of γ-radiation) or sham treatment. These samples then underwent LC-MS-based metabolic profiling to elucidate the delayed metabolic consequences of radiation exposure. (**B**) A three-dimensional PCA plot showing separation between radiation- and sham-treated groups at six months post-irradiation. (**C**) Volcano plot displaying dysregulated metabolites in mouse plasma at six months post-irradiation. In the plot, black dots represent metabolites that were not changed significantly; green dots represent metabolites with a significant fold change (1.5); blue dots represent metabolites with a significant *p*-value (<0.05); and red dots are used to annotate metabolites with a fold change of at least 1.5 as well as *p* < 0.05.

C57BL/6N WT (*n* = 20 males and *n* = 13 females) and transgenic APCHi mice (*n* = 17 males and *n* = 17 females) were subjected to *γ*-radiation at a single dose of 9.5 Gy with both hindlegs shielded from IR to allow long-term survival. Six-month survival in the mice with hind leg shielding was 100%. Plasma samples were obtained at 6 months after IR exposure and subjected to LC-MS-based metabolomic profiling (Figure 1A). Preprocessing of untargeted profiling data using eXtensible Computational Mass Spectrometry (XCMS) resulted in 3258 and 904 features detected in electrospray positive and negative ionization modes, respectively. Data normalization and log transformation were performed prior to univariate analysis. Binary group comparisons between sham and irradiated plasma samples resulted in the selection of 345 significantly dysregulated features based on a fold-change cut-off of >2 and FDR-adjusted *p*-value less than 0.05, and these dysregulated features were further selected for validation using fragment-based tandem mass spectrometry (MS/MS). Of the features selected for MS/MS validation, a subset of 48 metabolites were validated using fragmentation pattern matching by NIST (National Institute of Standards and Technology) or METLIN (Scripps Institute, La Jolla, CA, USA) databases; this methodology has been used by several research groups [12,13] (Supplementary Tables S1 and S2).

An in-house multiple reaction monitoring (MRM) system was developed based on quantitative metabolomics and lipidomic analytical methodologies and used for the identification and quantification of metabolites that exhibited IR-induced alteration in their abundance. Data processing followed by quality control measures provided us with 447 reliable metabolites, of which 63 were observed to be significantly dysregulated (fold-change cut-off of >1.5 and *p*-value < 0.05) for binary group comparisons between sham and irradiated animals within each genotype (Supplementary Table S3).

### 3.2. Exposure to γ-Radiation Induces Robust Changes in Mice

**Figure 2:**
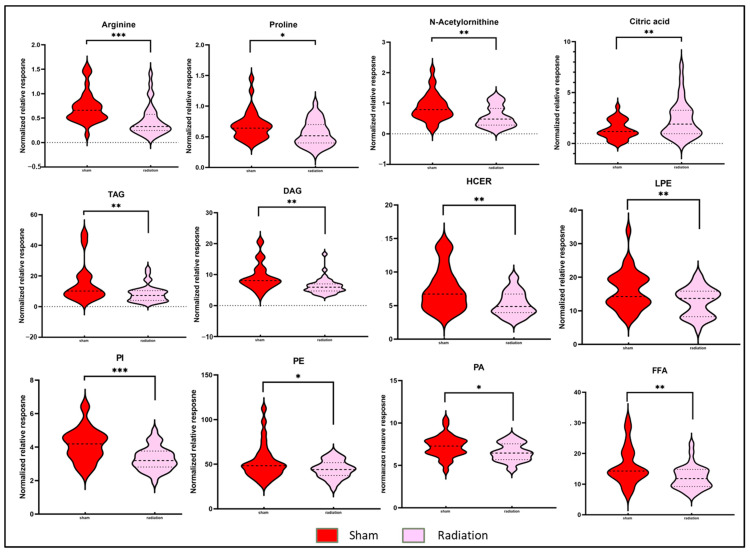
Radiation modulated plasma metabolic profiles six months after exposure. Violin plots illustrating the abundance of broad classes of lipids and small molecules between C57BL/6N mice in the sham condition and C57BL/6N mice in the irradiated condition six months post-irradiation. The dotted lines represent statistical markers: the center line represents the median, while the top and bottom lines represent the third and first quartiles, respectively. *: *p* ≤ 0.05, **: *p* ≤ 0.01 and ***: *p* ≤ 0.001.

To delineate the alterations in the plasma metabolic profiles at 6 months post-irradiation, we first compared the irradiated and sham-treated WT mice, irrespective of gender. Principal component analysis (PCA) dictated the distinctive plasma profile for irradiated mice compared to the sham group (Figure 1B). A volcano plot (Figure 1C) was used to visualize the differential abundance of significant (*p*-value < 0.05) metabolites. A total of 36 metabolites were observed as significantly dysregulated in radiation-exposed mice (Supplementary Table S3). Notable downregulations across several classes of lipids, such as fatty acyls, glycerophospholipids, glycerolipids, and sphingolipids, are reflective of radiation-induced dyslipidemia. A few representative small molecules and lipid species from each class are shown in Figure 2 and were selected based on their fold change and *p*-value when comparing irradiated mice plasma to the sham group. The subsided levels of amino acids, such as N-acetyl-ornithine, arginine, and proline, are suggestive of altered arginine (Arg) biosynthesis (Accessed on 10 January 2023; https://www.kegg.jp/kegg-bin/show_pathway?ko00220+K01429). Altered Arg levels could also be indicative of perturbations in the urea cycle. Arg is reported to mitigate the radiation-induced host immune dysfunction [14]. Tryptophan (Trp) is a product of intestinal microbiota, and the dysregulated Trp levels could be a consequence of early damage to the gut tissue [15]. Further, elevated citrate levels could be indicative of an altered TCA cycle [16]. Kyoto Encyclopedia of Genes and Genomes (KEGG) pathway analysis revealed perturbations in several pathways, including glycerophospholipid metabolism, linoleic acid metabolism, arginine, and proline metabolism after exposure to IR.

### 3.3. Gender-Based Metabolic Changes following 9.5 Gy γ-Radiation Exposure

**Figure 3:**
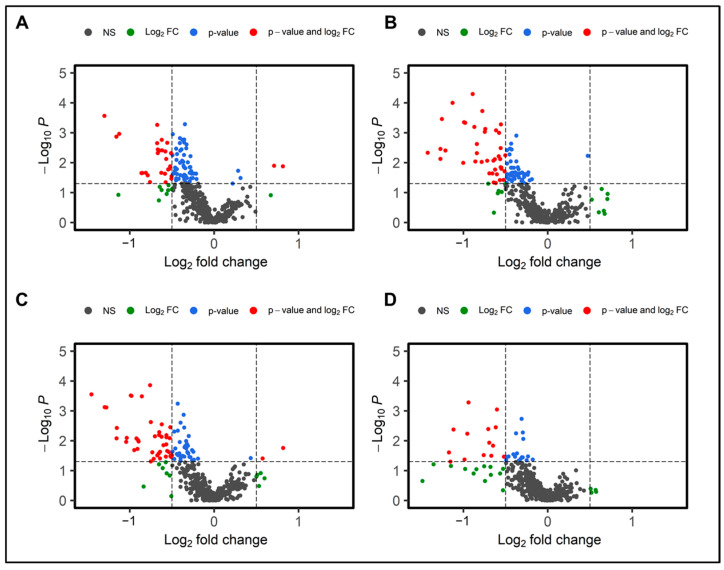
Radiation-induced metabolic alterations are modulated by gender and genotype. Dysregulated metabolites are visualized as volcano plots six months post-irradiation comparing sham and irradiated C57BL/6N male mice (**A**); sham and irradiated C57BL/6N female mice (**B**); sham and irradiated wild-type mice (**C**); and sham and irradiated APCHi mice (**D**).

Next, gender- and genotype-dependent metabolic alterations were examined. Volcano plots portrayed in Figure 3A–D describe the distinctive metabolic phenotypes in WT and APCHi, as well as gender-based modulation in host response.

Gender-based data analysis revealed noteworthy differences in the plasma metabolic profiles upon radiation exposure. Comparative sub-cohort analyses of irradiated versus sham mice showed significant dysregulation (fold change > 1.5 and *p*-value < 0.05) of 48 and 28 metabolites in female and male mice, respectively (Supplementary Tables S2 and S3). Downregulation of glycerophospholipids and fatty acyls, and metabolites such as Arg, tyrosine, prostaglandin E1 ethanolamide, and some indole metabolites were common to both male and female irradiated cohorts in comparison to their sham counterparts. Low tyrosine levels may indicate renal injury, which is a well-known metabolic alteration post radiation exposure [17]. Along with Trp, the metabolic disturbances in indole-3-lactic acid (I3LA), indole-3-propionic acid (I3PA), and tryptophol (indole-3-ethanol (IEA)) levels indicate disturbances in the gut microbiota-promoted shikimate pathway [18,19,20]. The upregulation of lathosterol, which is a precursor for cholesterols, also overlapped in both genders post-IR. Radiation-induced gut damage could result in bile-salt malabsorption and trigger enhanced hepatic production of cholesterol precursors. Bile salts are sterol metabolites, and dysregulations in cholesterol levels are associated with an increased risk of cancer and cardiovascular diseases [21]. N-Oleoyl-L-serine, an endogenous orphan lipid that mediates energy homeostasis and bone metabolism, was also upregulated in both genders.

**Figure 4:**
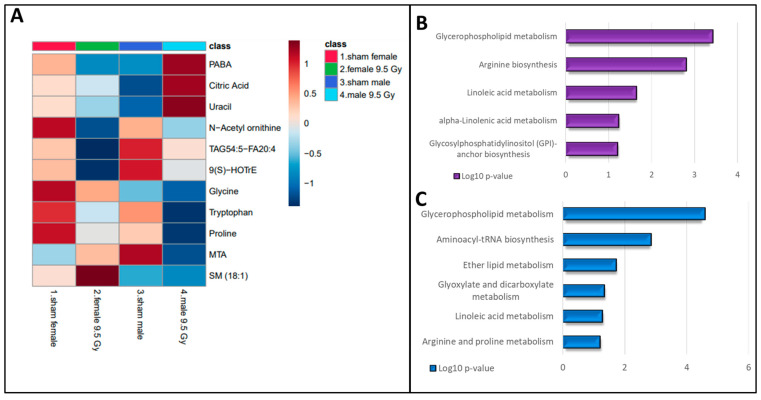
Radiation-induced metabolic alterations stratified by gender. Heatmap showing the gender-modulated differential abundance of metabolites at 6 months post-irradiation in C57BL/6N mouse plasma (**A**). KEGG pathway analysis was performed on plasma metabolites that showed significant changes in response to radiation exposure. Radiation-induced pathway dysregulation in female mice (**B**) and male mice (**C**) 6 months post-irradiation.

Conversely, some metabolites showed gender-specific modulation in radiation response. For example, the downregulated levels of amino acids (tryptophan, glycine, and proline) and upregulations in metabolites such as citric acid, 4-aminobenzoic acid (PABA), and uracil were observed to be specific to male mice. An altered abundance of purine metabolism intermediates and uracil is suggestive of perturbed nucleic acid biosynthesis. Likewise, a decrease in levels of TriAcylGlycerols (TAGs), N-acetylornithine, 9(S)-HOTrE, and an increase in sphingomyelin (18:1) were unique for irradiated female mice (Figure 4A). Dysregulated dopamine levels could be attributed to lower tyrosine levels that could potentially impact the functions of the neuronal system. The post-irradiation increase in GSH levels could be in response to combat enhanced oxidative stress burden. Pathway analysis showed dysregulation in glycerophospholipid, and the linoleic acid metabolism pathways were common to both genders after IR exposure. However, perturbations in Arg biosynthesis were found unique to female mice, while alterations in arginine and proline metabolism were specific to male mice (Figure 4B,C).

### 3.4. Genotype Specific Metabolic Changes following 9.5 Gy γ-Radiation Exposure

Next, the modulation of radiation response by the genotypic makeup of the mice was studied. Gender-independent binary comparisons were performed between the metabolic profiles of the 9.5 Gy irradiated APCHi group and the respective sham irradiated animals. Radiation-induced alterations in APCHi mice were then analyzed alongside the phenotyping changes exhibited by irradiated WT mice to assess differences in radiation response.

**Figure 5:**
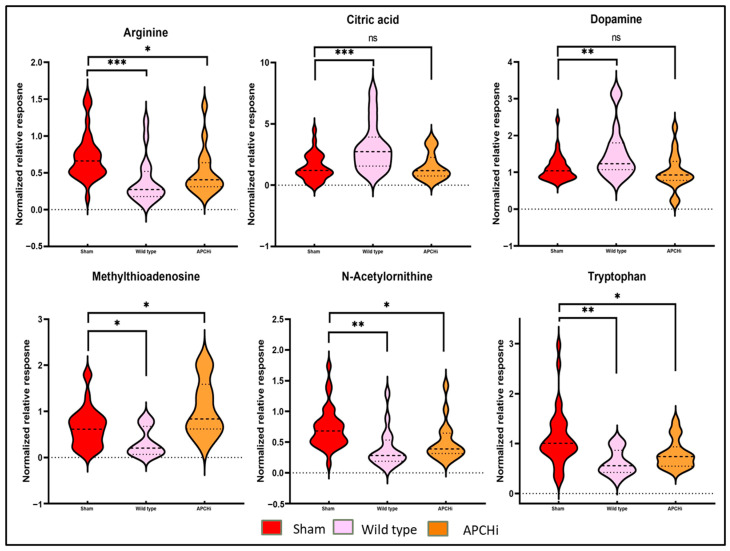
Mitigation of radiation toxicity in APCHi mice. Metabolic profiles of plasma samples of sham C57BL/6N and APCHi mice (combined for the analysis) were compared to 9.5 Gy of γ-irradiated C57BL/6N and APCHi mice six months post-treatment to see the mitigation effect of radiation toxicity by APC. LC-MS-based metabolic profiles of six selected metabolites are illustrated as Violin plots. The violin plots show a normalized relative abundance of mentioned metabolites between the sham condition, the C57BL/6N group, and the APCHi group 6 months post-irradiation. The dotted lines represent statistical markers: the center line represents the median, while the top and bottom lines represent the third and first quartiles, respectively. ns: *p* > 0.05, *: *p* ≤ 0.05, **: *p* ≤ 0.01, and ***: *p* ≤ 0.001.

The lipid classes, such as phospholipids (Phosphatidic Acids (PAs), phosphoethanolamine (PEs), LysoPhosphatidic Acids (LPAs), and free fatty acids (FFAs), which were found to be downregulated in irradiated WT mice, showed near normal abundance in the irradiated APCHi mice. Similarly, APCHi mice showed fewer IR-induced perturbations for a variety of small molecules, including amino acids (Figure 5). Amino acids are considered key regulators in maintaining vascular homeostasis by modulating EC proliferation, migration survival, and function [22,23]. Near normal levels of citrate in APCHi mice suggest a normalized TCA cycle. Upregulation of the TCA cycle plays a pivotal role in oncogenesis and inflammation [24]. Xanthurenic acid (XA) has been reported as a novel vasoactive compound, and its formation is a key event in the pathophysiology of inflammation-induced hypotension [25]. XA, which is a potential biomarker of radiation exposure and possibly renal failure [26,27,28], showed dysregulated abundance post-IR; however, it exhibited near-sham levels in APCHi mice. Subsided hypoxanthine levels in APCHi mice are suggestive of improved nucleic acid biosynthesis compared to irradiated WT mice. Also, higher levels of hypoxanthine are known to induce endothelial dysfunction through reactive oxygen species (ROS) production [29].

**Figure 6:**
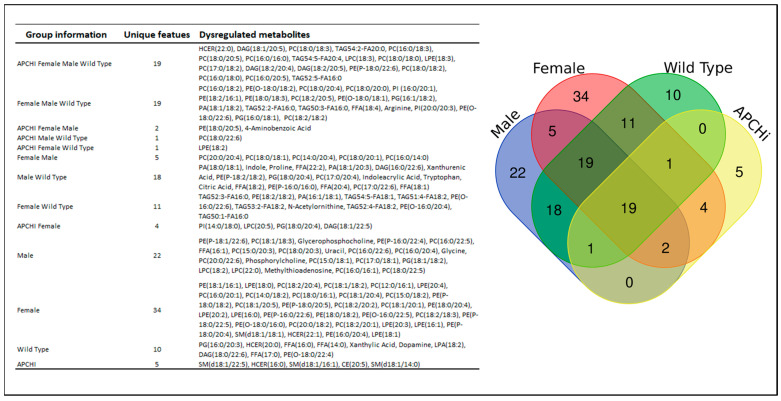
C57BL/6N WT (*n* = 20 males and *n* = 13 females) and transgenic APCHi mice (*n* = 17 males and *n* = 17 females) were subjected to γ-radiation at a single dose of 9.5 Gy. Plasma samples were obtained at 6 months after IR exposure and subjected to LC-MS-based metabolomic profiling. Gender- and genotype-dependent binary comparisons were performed between the metabolic profiles of the 9.5 Gy irradiated group with the respective sham irradiated animals and were summarized as a Venn diagram for unique radiation-induced metabolic changes specific to each gender and genotype.

The Venn diagram in Figure 6 details the significantly dysregulated metabolites, common and unique, from all binary group comparisons in the study.

## 4. Discussion

Accidental or intentional exposure to IR is a major public health concern that requires substantial efforts to manage and treat exposed individuals. Here, we utilized a metabolic phenotyping approach to characterize the molecular response to irradiation (9.5 Gy γ-radiation) and its modulation by genotype and gender using C57BL/6N and APCHi on the C57BL/6N background as mouse models. Plasma samples collected six months after exposure from male and female cohorts of mice were characterized using LC-MS-based metabolomics and lipidomics. Several lipidomic perturbations were identified, supporting existing evidence that significant dyslipidemia is a delayed effect of IR and may be used as an indicator of radiation exposure [24]. Additional long-term studies also indicate that lipid measures are appropriate biomarkers for DEARE [25]. Downregulation of glycerophospholipids may be an indication of chronic oxidative stress in irradiated mice [30]. Biosynthesis and degradation of phosphatidylcholines (PCs) are considered necessary for cell cycle progression, and the dysregulation of PC metabolism has been identified during apoptosis [31,32,33]. Glycerophospholipids are involved in several essential physiological activities, so their dysregulation may be an indicator of radiation-induced injury to several organs, such as the kidney, brain, and heart [34,35]. Subsided levels of free fatty acids following IR exposure could be a cellular response as a protective mechanism [36].

Disruption of amino acid metabolism following IR is well documented [37]. The decreased levels of Arg may affect physiological functions like cell proliferation, survival, and protein synthesis. Arg is a precursor of nitric oxide (NO), which is the most important endothelium-derived vasodilator molecule capable of promoting vascular health. However, ROS-promoted NO breakdown is the primary cause of endothelial dysfunction. Radiation-induced immune dysfunction is reported to be reversed by L-arginine [14]. Since Arg is an intermediate in the urea cycle, altered Arg levels could be indicative of a perturbed urea cycle and have impacts on downstream pathways such as creatine synthesis. The significance of arginine as a regulator of the mechanistic target of the rapamycin complex 1 (mTORC1) pathway lies in its role in mediating cell growth, proliferation, and metabolism. mTORC1 acts as a central hub in the cell, integrating signals from various sources, including nutrients, growth factors, and energy levels [38,39]. Arginine, an amino acid, is known to influence mTORC1 activity, thereby affecting cellular processes crucial for growth and proliferation [39]. Additionally, it suggests that mTOR inhibition (with rapamycin or its analogs), combined with radiation therapy, could potentially enhance the efficacy of cancer treatment by increasing radiation sensitivity [40]. Activation of mTORC1 in pericryptal mesenchymal cells through the induction of Insulin-like Growth Factor-1 (IGF-1) following radiation injury has been reported by Bohin et al. This finding suggests a potential mechanism by which radiation injury triggers cellular responses mediated by IGF-1, ultimately leading to mTORC1 activation in the mesenchymal cells surrounding the crypts [41]. Indeed, Wang et al. reported that mTORC1 signaling is activated following irradiation and plays a crucial role in facilitating the timely regeneration of the transient amplifying cell (TAC) pool within hair follicles. This underscores the importance of mTORC1 signaling in tissue regeneration and repair following radiation-induced damage, particularly in the context of hair follicle regeneration [42]. Near-normal levels of arginine observed in APCHi mice indicate that APC might confer radiation resistance by activating mTORC1. Future studies will be required to understand the mechanism of mTORC1 activation by APC.

Alterations in Trp metabolism are associated with many pathological states, such as gastrointestinal disorders, including inflammatory bowel diseases (IBD) and irritable bowel syndrome (IBS) [15,43]. Radiation-induced changes in the tryptophan/kynurenine ratios typically manifest as inflammation, renal failure, and cancer [27,44]. The TCA cycle is a hub for generating cellular energy, and the precursors for these biosynthetic pathways are known to modulate different aspects of cancer progression [45]. Upregulation of serum lathosterol levels, as observed in this study, is reported to cause perturbations in whole-body cholesterol synthesis [46]. Overall, the WT mice in this study were observed to exhibit IR-induced disruptions in several metabolic pathways relating to lipid metabolism, amino acid/protein metabolism, gut metabolome, energy metabolism, cholesterol, and nucleic acids biosynthesis that could potentially manifest to disorders relating to kidney damage, urea cycle, DNA damage or GIT injury.

Our analysis also revealed that the response to IR is affected by gender. The observed gender-specific alterations in metabolite abundance provide intriguing insights into the metabolic responses of male and female mice to irradiation. The downregulations of amino acids (tryptophan and proline) and upregulations in PABA and uracil were observed to be unique to male mice. Tryptophan serves as a precursor for serotonin and melatonin, neurotransmitters involved in mood regulation and sleep–wake cycles, respectively. Therefore, alterations in tryptophan levels could potentially impact neurobehavioral functions in male mice post-irradiation [47,48]. The role of proline in various physiological processes, including collagen formation, antioxidant defense, and cell signaling, is well-established in the scientific literature [49,50]. PABA may possess antioxidant properties and could potentially protect against oxidative stress-induced damage [51]. We observed an increase in sphingomyelin (18:1) in irradiated female mice. Sphingomyelin metabolism is tightly regulated and involves various enzymes and lipid transporters. Dysregulation of sphingomyelin metabolism has been implicated in several diseases, including neurodegenerative disorders and cardiovascular diseases [52,53]. Altogether, our findings suggest that sex plays a significant role in the response to IR. Further research is needed to understand the underlying mechanisms of these sex differences.

The role of APC-modulated protective effects in long-term radiation toxicity is still largely unknown, and further exploration is required. In this study, higher endogenous levels of APC were found to partially normalize post-IR plasma metabolic profiles, possibly indicating the alleviation of radiation injury in the irradiated APCHi group. Vascular phosphatidylethanolamine levels are believed to be a marker for endothelial dysfunction associated with hypertension [54]. Since Arg is known to improve EC functions through the promotion of NO production, the near sham levels of Arg and the related amino acids are reflective of normal Arg biosynthesis [14] and alleviated EC function. Also, observance of normal tryptophan abundance could be indicative of healthy intestine functions. In our study, APC was found to prevent TCA cycle irregularities, as indicated by normal citrate levels in the irradiated APCHi cohort compared to the sham cohort. Citrate can act as a metabolic regulator and is involved in numerous physiological and pathophysiological processes such as inflammation, cancer, insulin secretion, and neurological disorders [55,56,57]. Elevated hypoxanthine levels could enhance ROS production and induce endothelial dysfunction, thus leading to vascular complications [29]. However, near sham hypoxanthine levels as observed in APCHi animals is suggestive of ameliorated endothelial functions. Collectively, these findings suggest that the APCHi genotype imparts attenuation of metabolic perturbations caused by radiation exposure, which may be attributed to vascular endothelial repair.

Our prior study demonstrating metabolic alterations associated with radiation-induced cardiac injury, particularly showcasing differences in trends between APCHi and WT mice at 6 months after exposure to 9.5 Gy, indicates that there are distinct pathways affected by radiation exposure [9]. These findings suggest that perturbations in pathways could potentially be leveraged to mitigate the effects of DEARE on late-responding organ systems. Overall, these findings underscore the importance of elucidating the molecular pathways involved in radiation-induced tissue damage and highlight the potential for targeted therapeutic strategies to alleviate the late effects of radiation exposure on organ systems.

## 5. Conclusions

In conclusion, plasma metabolic profiles were used to characterize DEARE signatures in mice at 6 months post-IR. Our data show that increased circulating levels of endogenous APC constituted in mice normalized some IR-induced metabolic perturbations. Additional studies will be required to test the administration of APC as a mitigator of late radiation injuries and identify biological mechanisms by which APC may have different effects on certain DEARE in males compared to females.

## Data Availability

All data generated or analyzed during this study are included in this published article (and its Supplementary Information Files).

## Supplementary Materials

The following supporting information can be downloaded at: https://www.mdpi.com/article/10.3390/metabo14050245/s1, Table S1. Tandem MS validations for dysregulated metabolites in mice plasma, Table S2. List of dysregulated metabolites following exposure to 9.5 Gy of gamma-radiation, 6 months’ post irradiation, Table S3. List of dysregulated metabolites and lipids upon exposure to 9.5 Gy of gamma radiation, 6 months’ post irradiation.

## References

[1] Unthank J.L., Miller S.J., Quickery A.K., Ferguson E.L., Wang M., Sampson C.H., Chua H.L., DiStasi M.R., Feng H., Fisher A. (2015). Delayed Effects of Acute Radiation Exposure in a Murine Model of the H-ARS: Multiple-Organ Injury Consequent to <10 Gy Total Body Irradiation. Health Phys..

[2] Wang J., Boerma M., Fu Q., Hauer-Jensen M. (2007). Significance of endothelial dysfunction in the pathogenesis of early and delayed radiation enteropathy. World J. Gastroenterol..

[3] Guipaud O., Jaillet C., Clement-Colmou K., Francois A., Supiot S., Milliat F. (2018). The importance of the vascular endothelial barrier in the immune-inflammatory response induced by radiotherapy. Br. J. Radiol..

[4] Esmon C.T., Taylor F.B., Snow T.R. (1991). Inflammation and coagulation: Linked processes potentially regulated through a common pathway mediated by protein C. Thromb. Haemost..

[5] Mosnier L.O., Gale A.J., Yegneswaran S., Griffin J.H. (2004). Activated protein C variants with normal cytoprotective but reduced anticoagulant activity. Blood.

[6] Bansal S., Bansal S., Fish B.L., Li Y., Xu X., Fernandez J.A., Griffin J.H., Himburg H.A., Boerma M., Medhora M. (2023). Analysis of the urinary metabolic profiles in irradiated rats treated with Activated Protein C (APC), a potential mitigator of radiation toxicity. Int. J. Radiat. Biol..

[7] Isermann B., Vinnikov I.A., Madhusudhan T., Herzog S., Kashif M., Blautzik J., Corat M.A.F., Zeier M., Blessing E., Oh J. (2007). Activated protein C protects against diabetic nephropathy by inhibiting endothelial and podocyte apoptosis. Nat. Med..

[8] van Mens T.E., Liang H.H., Basu S., Hernandez I., Zogg M., May J., Zhan M., Yang Q., Foeckler J., Kalloway S. (2017). Variable phenotypic penetrance of thrombosis in adult mice after tissue-selective and temporally controlled Thbd gene inactivation. Blood Adv..

[9] Sridharan V., Johnson K.A., Landes R.D., Cao M., Singh P., Wagoner G., Hayar A., Sprick E.D., Eveld K.A., Bhattacharyya A. (2021). Sex-dependent effects of genetic upregulation of activated protein C on delayed effects of acute radiation exposure in the mouse heart, small intestine, and skin. PLoS ONE.

[10] Johnson C.H., Ivanisevic J., Siuzdak G. (2016). Metabolomics: Beyond biomarkers and towards mechanisms. Nat. Rev. Mol. Cell Biol..

[11] Salek R.M., Maguire M.L., Bentley E., Rubtsov D.V., Hough T., Cheeseman M., Nunez D., Sweatman B.C., Haselden J.N., Cox R.D. (2007). A metabolomic comparison of urinary changes in type 2 diabetes in mouse, rat, and human. Physiol. Genom..

[12] De Leoz M.L.A., Simon-Manso Y., Woods R.J., Stein S.E. (2019). Cross-Ring Fragmentation Patterns in the Tandem Mass Spectra of Underivatized Sialylated Oligosaccharides and Their Special Suitability for Spectrum Library Searching. J. Am. Soc. Mass. Spectrom..

[13] Cooper B.T., Yan X., Simon-Manso Y., Tchekhovskoi D.V., Mirokhin Y.A., Stein S.E. (2019). Hybrid Search: A Method for Identifying Metabolites Absent from Tandem Mass Spectrometry Libraries. Anal. Chem..

[14] Shukla J., Chatterjee S., Thakur V.S., Premachandran S., Checker R., Poduval T.B. (2009). L-Arginine reverses radiation-induced immune dysfunction: The need for optimum treatment window. Radiat. Res..

[15] Agus A., Planchais J., Sokol H. (2018). Gut Microbiota Regulation of Tryptophan Metabolism in Health and Disease. Cell Host Microbe.

[16] Martinez-Reyes I., Chandel N.S. (2020). Mitochondrial TCA cycle metabolites control physiology and disease. Nat. Commun..

[17] Nepomuceno G., Junho C.V.C., Carneiro-Ramos M.S., da Silva Martinho H. (2021). Tyrosine and Tryptophan vibrational bands as markers of kidney injury: A renocardiac syndrome induced by renal ischemia and reperfusion study. Sci. Rep..

[18] Zhang F.L., Chen X.W., Wang Y.F., Hu Z., Zhang W.J., Zhou B.W., Ci P.F., Liu K.X. (2023). Microbiota-derived tryptophan metabolites indole-3-lactic acid is associated with intestinal ischemia/reperfusion injury via positive regulation of YAP and Nrf2. J. Transl. Med..

[19] Konopelski P., Mogilnicka I. (2022). Biological Effects of Indole-3-Propionic Acid, a Gut Microbiota-Derived Metabolite, and Its Precursor Tryptophan in Mammals’ Health and Disease. Int. J. Mol. Sci..

[20] Scott S.A., Fu J., Chang P.V. (2020). Microbial tryptophan metabolites regulate gut barrier function via the aryl hydrocarbon receptor. Proc. Natl. Acad. Sci. USA.

[21] Hageman J., Herrema H., Groen A.K., Kuipers F. (2010). A role of the bile salt receptor FXR in atherosclerosis. Arter. Thromb. Vasc. Biol..

[22] Li M., Wu Y., Ye L. (2022). The Role of Amino Acids in Endothelial Biology and Function. Cells.

[23] Pi X., Xie L., Patterson C. (2018). Emerging Roles of Vascular Endothelium in Metabolic Homeostasis. Circ. Res..

[24] Scagliola A., Mainini F., Cardaci S. (2020). The Tricarboxylic Acid Cycle at the Crossroad Between Cancer and Immunity. Antioxid. Redox Signal..

[25] Fazio F., Carrizzo A., Lionetto L., Damato A., Capocci L., Ambrosio M., Battaglia G., Bruno V., Madonna M., Simmaco M. (2017). Vasorelaxing Action of the Kynurenine Metabolite, Xanthurenic Acid: The Missing Link in Endotoxin-Induced Hypotension?. Front. Pharmacol..

[26] Malina H.Z., Richter C., Mehl M., Hess O.M. (2001). Pathological apoptosis by xanthurenic acid, a tryptophan metabolite: Activation of cell caspases but not cytoskeleton breakdown. BMC Physiol..

[27] Pawlak D., Tankiewicz A., Mysliwiec P., Buczko W. (2002). Tryptophan metabolism via the kynurenine pathway in experimental chronic renal failure. Nephron.

[28] Goudarzi M., Mak T.D., Chen C., Smilenov L.B., Brenner D.J., Fornace A.J. (2014). The effect of low dose rate on metabolomic response to radiation in mice. Radiat. Environ. Biophys..

[29] Kim Y.J., Ryu H.M., Choi J.Y., Cho J.H., Kim C.D., Park S.H., Kim Y.L. (2017). Hypoxanthine causes endothelial dysfunction through oxidative stress-induced apoptosis. Biochem. Biophys. Res. Commun..

[30] Schiller J., Fuchs B., Arnhold J., Arnold K. (2003). Contribution of reactive oxygen species to cartilage degradation in rheumatic diseases: Molecular pathways, diagnosis and potential therapeutic strategies. Curr. Med. Chem..

[31] Leal-Esteban L.C., Fajas L. (2020). Cell cycle regulators in cancer cell metabolism. Biochim. Biophys. Acta Mol. Basis Dis..

[32] Saito R.F., Andrade L.N.S., Bustos S.O., Chammas R. (2022). Phosphatidylcholine-Derived Lipid Mediators: The Crosstalk between Cancer Cells and Immune Cells. Front. Immunol..

[33] Ridgway N.D. (2013). The role of phosphatidylcholine and choline metabolites to cell proliferation and survival. Crit. Rev. Biochem. Mol. Biol..

[34] Wirthensohn G., Beck F.X., Guder W.G. (1987). Role and regulation of glycerophosphorylcholine in rat renal papilla. Pflug. Arch..

[35] Farooqui A.A., Horrocks L.A., Farooqui T. (2000). Glycerophospholipids in brain: Their metabolism, incorporation into membranes, functions, and involvement in neurological disorders. Chem. Phys. Lipids.

[36] Munir R., Lisec J., Swinnen J.V., Zaidi N. (2019). Lipid metabolism in cancer cells under metabolic stress. Br. J. Cancer.

[37] Laiakis E.C., Strassburg K., Bogumil R., Lai S., Vreeken R.J., Hankemeier T., Langridge J., Plumb R.S., Fornace A.J., Astarita G. (2014). Metabolic phenotyping reveals a lipid mediator response to ionizing radiation. J. Proteome Res..

[38] Carroll B., Maetzel D., Maddocks O.D., Otten G., Ratcliff M., Smith G.R., Dunlop E.A., Passos J.F., Davies O.R., Jaenisch R. (2016). Control of TSC2-Rheb signaling axis by arginine regulates mTORC1 activity. eLife.

[39] Kim J., Guan K.L. (2019). mTOR as a central hub of nutrient signalling and cell growth. Nat. Cell Biol..

[40] Phan M., Kim C., Mutsaers A., Poirier V., Coomber B. (2022). Modulation of mTOR signaling by radiation and rapamycin treatment in canine mast cell cancer cells. Can. J. Vet. Res..

[41] Bohin N., McGowan K.P., Keeley T.M., Carlson E.A., Yan K.S., Samuelson L.C. (2020). Insulin-like Growth Factor-1 and mTORC1 Signaling Promote the Intestinal Regenerative Response After Irradiation Injury. Cell. Mol. Gastroenterol. Hepatol..

[42] Wang W.H., Chien T.H., Fan S.M., Huang W.Y., Lai S.F., Wu J.T., Lin S.J. (2017). Activation of mTORC1 Signaling is Required for Timely Hair Follicle Regeneration from Radiation Injury. Radiat. Res..

[43] Burr R.L., Gu H., Cain K., Djukovic D., Zhang X., Han C., Callan N., Raftery D., Heitkemper M. (2019). Tryptophan Metabolites in Irritable Bowel Syndrome: An Overnight Time-course Study. J. Neurogastroenterol. Motil..

[44] Suzuki Y., Suda T., Furuhashi K., Suzuki M., Fujie M., Hahimoto D., Nakamura Y., Inui N., Nakamura H., Chida K. (2010). Increased serum kynurenine/tryptophan ratio correlates with disease progression in lung cancer. Lung Cancer.

[45] Eniafe J., Jiang S. (2021). The functional roles of TCA cycle metabolites in cancer. Oncogene.

[46] Duane W.C. (1995). Serum lathosterol levels in human subjects reflect changes in whole body cholesterol synthesis induced by lovastatin but not dietary cholesterol. J. Lipid Res..

[47] Richard D.M., Dawes M.A., Mathias C.W., Acheson A., Hill-Kapturczak N., Dougherty D.M. (2009). L-Tryptophan: Basic Metabolic Functions, Behavioral Research and Therapeutic Indications. Int. J. Tryptophan Res..

[48] Jenkins T.A., Nguyen J.C., Polglaze K.E., Bertrand P.P. (2016). Influence of Tryptophan and Serotonin on Mood and Cognition with a Possible Role of the Gut-Brain Axis. Nutrients.

[49] Phang J.M., Liu W., Zabirnyk O. (2010). Proline metabolism and microenvironmental stress. Annu. Rev. Nutr..

[50] Hu C.A., Donald S.P., Yu J., Lin W.W., Liu Z., Steel G., Obie C., Valle D., Phang J.M. (2007). Overexpression of proline oxidase induces proline-dependent and mitochondria-mediated apoptosis. Mol. Cell. Biochem..

[51] Sirota T.V., Lyamina N.E., Weisfeld L.I. (2017). The Antioxidant properties of para-Aminobenzoic acid and its sodium salt. Biophysics.

[52] Hannun Y.A., Obeid L.M. (2018). Sphingolipids and their metabolism in physiology and disease. Nat. Rev. Mol. Cell Biol..

[53] Maceyka M., Spiegel S. (2014). Sphingolipid metabolites in inflammatory disease. Nature.

[54] Zhao M. (2015). Imaging Vascular Phosphatidylethanolamine.

[55] Icard P., Poulain L., Lincet H. (2012). Understanding the central role of citrate in the metabolism of cancer cells. Biochim. Et Biophys. Acta.

[56] Infantino V., Convertini P., Cucci L., Panaro M.A., Di Noia M.A., Calvello R., Palmieri F., Iacobazzi V. (2011). The mitochondrial citrate carrier: A new player in inflammation. Biochem. J..

[57] Cappello A.R., Guido C., Santoro A., Santoro M., Capobianco L., Montanaro D., Madeo M., Andò S., Dolce V., Aquila S. (2012). The mitochondrial citrate carrier (CIC) is present and regulates insulin secretion by human male gamete. Endocrinology.

