# Supplementary material for: Genetic Upregulation of Activated Protein C Mitigates Delayed Effects of Acute Radiation Exposure in the Mouse Plasma
Source: Metabolites. 2024 Apr 24;14(5):245. doi: 10.3390/metabo14050245 (PMC11122730; doi:10.3390/metabo14050245)
Supplement: Supplementary file 1 [file metabolites-14-00245-s001.zip › metabolites-2961431-supplementary.pdf]

Genetic upregulation of activated protein C mitigates delayed effects of acute radiation exposure in the mouse plasma

[$]Shivani Bansal [A], [$]Yaoxiang Li [A], Sunil Bansal [A], William Klotzbier [A] Baldev Singh [A], Meth Jayatilake [A], Vijayalakshmi Sridharan [B], Jose A. Fernández [C], John H. Griffin [C], Hartmut Weiler [D], Marjan Boerma [B], Amrita K Cheema [A, E]*

[A] Department of Oncology, Lombardi Comprehensive Cancer Center, Georgetown University Medical Center, Washington D.C., 20057, USA.

[B] Division of Radiation Health, Department of Pharmaceutical Sciences, , University of Arkansas for Medical Sciences, Little Rock, Arkansas, 72205, USA.

[C] Department of Molecular Medicine, Scripps Research Institute, La Jolla, CA 92037, USA.

[D] Versiti Blood Research Institute and the Medical College of Wisconsin, Milwaukee, Wisconsin, 53233, USA.

[E] Departments of Biochemistry, Molecular and Cellular Biology, Georgetown University Medical Center, Washington D.C, 20057, USA.

$: Both authors contributed equally.

* Correspondence: Amrita K Cheema

Professor; Georgetown University Medical Center, GC2 Pre-Clinical Science Building, 3900 Reservoir Road NW Washington DC 20057; Phone (202) 687-2756; Fax (202) 687-8860; e-mail: akc27@georgetown.edu

LC gradient and MS instrument parameters

Untargeted metabolomics

For untargeted metabolomics data acquisition, each sample (1 µL) was injected to a 1.7 µm, 2.1 mm × 50 mm Acquity BEH C18 column (Waters Corporation, MA, USA) using an Acquity UPLC system connected to an electrospray ion source coupled with a quadrupole time-of-flight mass spectrometer (ESI-Q-TOF, Xevo-G2S, Waters Corporation, MA, USA) operating in positive and negative ionization mode. The gradient mobile phases consisted of 100% water with 0.1% formic acid (solvent A), 100% acetonitrile with 0.1% formic acid (solvent B), 100% isopropanol with 0.1% formic acid (solvent C). Each sample injection was run for 13 minutes at a flow rate for 500 µL/min. The LC gradient conditions with a ramp curve of 6 at each step were as follows: Initial – 98% A, 2% B till 0.5 minutes; 4.0 minutes – 40% A, 60% B; 8.0 minutes – 2% A, 98% B till 9.0 minutes; 9.5 minutes – 11.8% B, 88.2% C till 11 minutes; 11.5 minutes - 50% A, 50% B, 12.0 minutes – 98% A, 2% B till 13 minutes. Positive mode had a capillary voltage of 2.75 kV, a sampling cone voltage of 30 V, and a source offset of 80 V. Negative mode had a capillary voltage of 2.75 kV, a sampling cone voltage of 20 V, and a source offset of 80 V. The desolvation gas flow was 600 L/h. and the temperature was set to 500 °C. The cone gas flow was 25 L/h, and the source temperature was 100 °C. The data were acquired in the sensitivity MS Mode with a scan time of 1.000 seconds, and inter-scan delay at 0.014 seconds. Real-time mass correction was applied using a solution of Leucine-Enkephalin (1.0 ng/ml) [M+H]+ (m/z 556.2771), [M−H] (m/z 554.2615) in 500 ml of a mixture containing acetonitrile: water (50:50) and 250 µL formic acid at an infusion rate of 20 µL/min utilizing the Waters Lockspray® interface. Before and after samples run, a mixture of six standards (acetaminophen: m/z 152.0712 [M+H]+/150.0555 [M−H]−, sulfaguanidine: m/z 215.0603 [M+H]+/213.0446 [M−H]−, sulfadimethoxine: m/z 311.0814 [M+H]+/309.0658 [M−H]−, Val-Tyr-Val: m/z 380.2185 [M+H]+/378.2029 [M−H]−, terfenadine: m/z 472.3216 [M+H]+ and leucine-enkephalin: m/z 556.2771 [M+H]+/554.2615 [M−H]−) were run to ensure mass accuracy during data acquisition (Supplementary Figure 3 and 4).

Targeted metabolomics

A binary solvent comprising of water with 0.1% formic acid (solvent A) and acetonitrile with 0.1% formic acid (solvent B) was used. The extracted metabolites were resolved at 0.2 mL/min flow rate starting with 100% of solvent A and hold for 2.1 minutes and moving to 5% of solvent A over a time period of 12 minute and hold for 1 minute and equilibrating to initial conditions over a time period of 7 minutes using auto sampler temperature 15 °C and oven temperature 30 °C. Source and gas setting for the method were as follow: curtain gas = 35, CAD gas = medium, ion spray voltage = 2500 V in positive mode and −4500 V in negative mode, temperature = 400 °C, nebulizing gas = 60 and heater gas = 70.

Targeted Lipidolomics

A binary solvent comprising of acetonitrile/water 95/5 with 10 mM ammonium acetate as solvent A and acetonitrile/water 50/50 with 10 mM ammonium acetate as solvent B was used for the resolution. Lipids were resolved at 0.7 mL/min flow rate, initial gradient conditions started with 100% of solvent A, shifting towards 99.9% of solvent A over a duration of 3 minutes, 94% of solvent A over a time period of 3 minutes and 25% of solvent A over a period of 4 minutes. Finally, washing with 100% of B for 6 minutes and equilibrating to initial conditions (100% of solvent A) over a time period of 6 minutes using auto sampler

temperature 15 °C and oven temperature 35 °C. Source and gas setting were as follow: curtain gas = 30, CAD gas = medium, ion spray voltage = 5.5 kV in positive mode and −4.5 kV in negative mode, temperature = 550 °C, nebulizing gas = 50 and heater gas = 60.

Data processing and statistical analysis

The quality and reproducibility of LC-MS data was ensured using several measures. The column was conditioned using a pooled QC sample that was prepared as a pooled aliquot of all samples in the study set. The QC sample was injected periodically (after every 10 sample injections) to monitor shifts in signal intensities and retention time as measures of reproducibility and ensure high quality data acquisition. A solvent blank sample was also run periodically to monitor and minimize the sample-to-sample carry-over. For targeted data, the detected metabolites were filtered using a signal/noise ratio >20:1 and retention time (RT) tolerance of 5 seconds criteria, to identify the reliable features. The details on LC gradient and mass spectrometry instrument parameters have been provided in the Supplementary information. Data pre-processing include normalization of the raw LC-MS data initially using internal standards. The detected metabolites/features (m/z_rt pairs) with more than 20% missing value were filtered out and with less than 20% of missing value were imputed by half of the minimum positive value in the original data. The features with more than 15% of coefficient of variance (CV) were also filtered out. The remaining high-quality features were normalized by Quality Control-Robust LOESS Signal Correction (QC-RLSC) followed by statistical analyses using R (version 4.0.1). For the 67 samples in the study set, the level of differential expression for each metabolite was calculated using an unpaired t-test, comparing sham versus irradiated plasma samples, constrained by p-value<0.05. We also compared gender and genotype based metabolic differences post irradiation in mice plasma. The identities of significantly dysregulated metabolites were confirmed using tandem mass spectrometry. Raw files from untargeted data were converted into MSP format with an in-house developed R package "msmsr" (Li et al., unpublished) and the NIST 2017 MS/MS spectra database. Mummichog v2.0, a Python package specifically designed for untargeted metabolomics was used for pathway analysis [12]. Mummichog v2.0 tests pathway enrichment patterns using permutations and computes the probability for involvement in each pathway. Figures were created using R and BioRender (www.BioRender.com).

Supplementary Table S1. Tandem MS validations for dysregulated metabolites in mice plasma

file_name	precursor_m/z_rt	Mode	Name	Synonyms	Class	Adduct	Level	CID
20200127_uo1_apc_plasma_metabolomics_MSMS_NEG_02501.CDF	313.0637_0.84	Negative	Salicylic acid β-D-O-glucuronide	Salicyl glucuronide		[M-H]-	B	313.06, 312.17, 295.22, 185l.01, 183.01, 179.08, 175.03, 137.02, 133.09, 129.06, 117.06, 99.01
20200127_uo1_apc_plasma_metabolomics_MSMS_NEG_03201.CDF	327.2307_7.71	Negative	1-(1Z-Octadecenyl)-2-(4Z,7Z,10Z,13Z,16Z,19Z-docosahexaenoyl)-sn-glycero-3-phosphoethanolamine	PE(P-18:0/22:6)	Lipid-PE	[M-H-C27H43O5]-	B	328.23, 327.23, 326.19, 327.18, 309.20, 283.24, 229.19, 191.17, 183.01, 177.16
20200127_uo1_apc_plasma_metabolomics_MSMS_NEG_05701.CDF	124.0034_0.69	Negative	Taurine	Taurine		[M-H]-	B	124.01, 106.98, 64.9705, 79.96
20200127_uo1_apc_plasma_metabolomics_MSMS_NEG_08901.CDF	728.557_10.73	Negative	1-(1Z-Octadecenyl)-2-(9Z-octadecenoyl)-sn-glycero-3-phosphoethanolamine	PE(P-18:0/18:1)	Lipid-PE	[M-H]-	B	731.55, 730.56, 729.57, 728.56, 466.31, 465.30, 460.27, 447.29, 283.24, 282.25, 281.25, 267.23, 196.03, 140.00
20200127_uo1_apc_plasma_metabolomics_MSMS_NEG_09601.CDF	850.56_10.13	Negative	1-Palmitoyl-2-docosahexaenoyl-sn-glycero-3-phosphocholine	PC(38:6) /PC(16:0/22:6)	Lipid-PC	[M+HCO2]-	A	850.56, 790.54, 552.31, 480.31, 327.23, 283.24, 255.23
20200124_uo1_apc_plasma_metabolomics_MSMS_POS_00901.CDF	120.0807_1.14	Positive	N-Acetyl-L-phenylalanine	N-Ac-Phe	Amino acid conjugate	[M+H-C3H4O3]+	B	121.08, 120.08, 119.07, 118.01, 103.05, 93.07, 91.05, 79.05
20200124_uo1_apc_plasma_metabolomics_MSMS_POS_01001.CDF	121.0282_5.41	Positive	4-Hydroxybenzoic acid	4-OH BnCOOH		[M+H-H2O]+	A	122.09, 111.04, 105.06, 95.04
20200124_uo1_apc_plasma_metabolomics_MSMS_POS_01101.CDF	121.0283_4.46	Positive	5-Methyl-5,6-Dihydrouracil	5,6-Dihydrothymine	Nitrogenous base	[M+H]+	C	129.06, 69.02
20200124_uo1_apc_plasma_metabolomics_MSMS_POS_01601.CDF	132.0663_0.82	Positive	N-Acetyl-L-alanine	N-Ac-L-alanine	Amino acid conjugate	[M+H]+	A	132.07, 90.05, 86.09
20200124_uo1_apc_plasma_metabolomics_MSMS_POS_02101.CDF	140.0681_0.64	Positive	N-Carboxyethyl-γ-aminobutyric acid	Spermidic acid	Amino acid conjugate	[M+H-2H2O]+	C	140.06, 116.97, 98.96, 98.06, 70.00
20200124_uo1_apc_plasma_metabolomics_MSMS_POS_02501.CDF	144.0805_2.01	Positive	3-(2-Hydroxyethyl)indole	1H-Indole-3-ethanol		[M+H-H2O]+	A	144.08, 143.07, 142.06, 128.05, 127.05, 118.06, 117.06, 115.05, 103.05, 91.05, 77.03
20200124_uo1_apc_plasma_metabolomics_MSMS_POS_03301.CDF	159.091_2.01	Positive	4-(1-Piperazinyl)-1H-indole1	4-(Piperazino)indole		[M+H-C2H5N]+	B	159.09, 157.03, 144.06, 142.06, 133.06, 132.06, 130.06, 117.05
20200124_uo1_apc_plasma_metabolomics_MSMS_POS_03601.CDF	162.1125_0.67	Positive	L-Carnitine	L-Carnitine	Carnitine	[M+H]+	A	162.11, 103.04, 102.09, 60.08
20200124_uo1_apc_plasma_metabolomics_MSMS_POS_03901.CDF	170.0597_2.01	Positive	DL-Indole-3-lactic acid	3-Indolyllactic acid		[M+H-2H2O]+	A	170.06, 142.06, 115.05
20200124_uo1_apc_plasma_metabolomics_MSMS_POS_04301.CDF	188.0706_2.01	Positive	L-Tryptophan	Tyr	Amino acid	[M+H-NH3]+	A	188.07, 170.06, 146.06, 118.06
20200124_uo1_apc_plasma_metabolomics_MSMS_POS_04401.CDF	190.0857_3.78	Positive	1H-Indole-3-propanoic acid	3-Indolepropionic acid	potent neuroprotective antioxidant	[M+H]+	A	190.09, 189.07, 172.07, 130.06
20200124_uo1_apc_plasma_metabolomics_MSMS_POS_04501.CDF	203.0528_0.64	Positive	DL-Indole-3-lactic acid	3-Indolyllactic acid		[M+H]+	A	206.00, 188.07, 170.06, 160.07, 146.06, 144.08, 132.08, 130.06, 118.06
20200124_uo1_apc_plasma_metabolomics_MSMS_POS_05901.CDF	261.2205_7.37	Positive	Pinolenic acid	Pinolenic acid	FFA	[M+H-H2O]+	C	261.21, 243.20, 217.19, 135.11, 109.11, 105.07, 81.07
20200124_uo1_apc_plasma_metabolomics_MSMS_POS_06901.CDF	277.215_5.83	Positive	9S-Hydroxy-10E,12Z,15Z-octadecatrienoic acid	9(S)-HOTrE	FFA	[M+H-H2O]+	C	277.18, 221.01, 109.10, 95.08, 81.07, 67.05
20200124_uo1_apc_plasma_metabolomics_MSMS_POS_07001.CDF	277.2155_6.96	Positive	Stearidonic acid	Moroctic acid C18:4	FFA	[M+H]+	C	277.216, 235.17, 133.10, 125.00, 123.11, 109.10, 107.08, 97.10, 95.08, 81.07, 67.05
20200124_uo1_apc_plasma_metabolomics_MSMS_POS_07201.CDF	279.2313_7.37	Positive	Pinolenic acid	Pinolenic acid	FFA	[M+H]+	B	279.23, 135.11, 133.10, 131.08, 123.11, 121.10, 119.05, 117.02, 95.08, 81.07, 79.05, 67.05
20200124_uo1_apc_plasma_metabolomics_MSMS_POS_07501.CDF	281.2471_7.84	Positive	10E,12Z-octadecadienoic acid	Conjugated linoleic acid (10E,12Z)	FFA	[M+H]+	B	281.14, 263.23, 245.22, 221.02, 123.11, 121.10, 119.08, 111.11, 109.10, 97.10, 95.08, 93.07
20200124_uo1_apc_plasma_metabolomics_MSMS_POS_07901.CDF	283.2631_8.35	Positive	trans-Vaccenic acid	(11E)-11-Octadecenoic acid	FFA	[M+H]+	B	265.25, 247.24, 209.18, 129.06, 125.00, 123.11, 121.10, 111.10, 109.10, 107.08, 93.07, 83.08, 81.06, 71.08, 69.07, 67.05
20200124_uo1_apc_plasma_metabolomics_MSMS_POS_08301.CDF	288.2527_4.79	Positive	Lauric acid diethanolamide	Alkamide LE		[M+H]+	C	288.25, 270.24, 109.10, 106.09, 95.08, 88.08, 81.07, 71.08, 60.04
20200124_uo1_apc_plasma_metabolomics_MSMS_POS_09301.CDF	305.2479_7.72	Positive	cis-5,8,11,14-Eicosatetraenoic acid	Arachidonic acid	FFA	[M+H]+	A	305.24, 304.23, 269.22, 259.20, 245.22, 221.15, 199.15, 193.12, 191.18, 189.13, 122.10, 120.09, 118.07, 109.10, 107.08
20200124_uo1_apc_plasma_metabolomics_MSMS_POS_09601.CDF	307.2631_8.06	Positive	5Z,11Z,14Z-Eicosatrienoic acid	5,6-EpETrE	FFA	[M+H]+	B	307.26, 289.25, 271.24, 123.12, 109.10, 81.07, 79.05, 67.05
20200124_uo1_apc_plasma_metabolomics_MSMS_POS_10101.CDF	319.2636_8.65	Positive	ω-3 Arachidonic acid methyl ester	ω-3 Arachidonic acid methyl ester		[M+H]+	B	319.15, 287.23, 161.13, 147.12, 123.11, 121.10, 119.08, 117.07 107.08, 97.10, 95.08, 93.07, 91.05
20200124_uo1_apc_plasma_metabolomics_MSMS_POS_10701.CDF	325.2111_7.86	Positive	Maresin 1	Maresin 1		[M+H-2H2O]+	A	325.21, 173.13, 171.11, 145.10, 145.10, 143.08, 141.07, 131.08, 129.07, 121.10, 119.08, 117.07, 95.08, 93.07, 91.04, 81.07, 79.05, 67.05
20200124_uo1_apc_plasma_metabolomics_MSMS_POS_10901.CDF	327.2322_7.25	Positive	10-hydroxy-4Z,7Z,11E,13Z,16Z,19Z-docosahexaenoic acid	(±)10-HDoHE	FFA	[M+H-H2O]+	A	327.23, 273.18, 267.21, 265.19, 259.17, 163.14, 59.11, 155.08, 149.13, 145.10, 143.10, 141.07, 139.07, 123.11, 119.08, 105.07, 95.08, 93.07, 81.07, 79.05
20200124_uo1_apc_plasma_metabolomics_MSMS_POS_11001.CDF	329.2086_4.29	Positive	5α-Dihydrocortisol	Hydrallostane	Cortisol Metabolite	[M+H-2H2O]+	B	329.25, 311.24, 283.20, 171.11, 169.10, 149.13, 145.10, 143.08, 143.08, 141.06, 133.10, 119.08, 117.07, 95.08, 91.04, 81.07, 79.05
20200124_uo1_apc_plasma_metabolomics_MSMS_POS_11101.CDF	329.2481_7.61	Positive	cis-5,8,11-Eicosatrienoic acid	cis-5,8,11-Eicosatrienoic acid	FFA	[M+Na]+	A	330.25, 329.25, 311.24, 293.23, 283.20, 269.22, 259.17, 201.16, 199.14, 197.12, 193.12, 187.15, 185.13, 185.13, 181.10, 179.11, 123.12, 121.10, 119.08, 109.10, 95.08, 93.07, 91.04, 81.07, 79.05
20200124_uo1_apc_plasma_metabolomics_MSMS_POS_13001.CDF	357.2789_5.57	Positive	Chenodeoxycholic acid	chenocholic acid	Bile Acid	[M+H-2H2O]+	A	357.27, 147.12, 145.10, 135.12, 123.11, 121.10, 119.08, 109.10, 107.08, 101.06, 97.10, 95.06, 93.07, 81.07, 79.05, 77.03
20200124_uo1_apc_plasma_metabolomics_MSMS_POS_14301.CDF	365.3182_7.28	Positive	7α,24(S)-Dihydroxycholesterol	7α,24(S)-Dihydroxycholesterol	Oxysterol	[M+H-3H2O]+	A	365.25, 309.28, 299.19, 217.19, 203.18, 201.16, 191.17, 187.14, 149.13, 147.12, 135.11, 123.11, 12110, 119.08, 109.10, 107.08, 105.07, 95.08, 81.07
20200124_uo1_apc_plasma_metabolomics_MSMS_POS_14701.CDF	369.3512_10.89	Positive	5α-Cholest-7-en-3β-ol	Lathosterol		[M+H-H2O]+	A	369.35, 368.34, 287.27, 259.24, 243.21, 215.18, 163.15, 161.13, 149.13, 147.12, 145.10, 135.12, 123.11, 109.10, 95.09, 93.07, 71.08, 67.05
20200124_uo1_apc_plasma_metabolomics_MSMS_POS_14801.CDF	369.3519_10.2	Positive	5α-Cholest-7-en-3β-ol	Lathosterol		[M+H-H2O]+	A	
20200124_uo1_apc_plasma_metabolomics_MSMS_POS_14901.CDF	370.2712_7.6	Positive	N-Arachidonylmaleimide	N-Arachidonylmaleimide	Monoacylglycerol lipase (MAGL) inhibitor	[M+H]+	C	370.28, 328.22, 189.16, 161.13, 151.15, 135.11, 121.10, 83.07, 81.07, 79.05, 69.05, 67.05
20200124_uo1_apc_plasma_metabolomics_MSMS_POS_15101.CDF	370.2949_5.1	Positive	N-Oleoyl-L-serine	N-Oleoyl-L-serine	Amino acid conjugate	[M+H]+	B	370.28, 149.13, 135.12, 123.11, 121.10, 97.10, 95.08, 93.07, 81.07, 79.05, 71.08, 67.05
20200124_uo1_apc_plasma_metabolomics_MSMS_POS_16201.CDF	380.2556_5.58	Positive	D-erythro-Sphingosine-1-phosphate	S1P	Lipid	[M+H]+	B	380.28, 362.25, 264.27
20200124_uo1_apc_plasma_metabolomics_MSMS_POS_16301.CDF	380.2846_7.99	Positive	Prostaglandin E1 ethanolamide	Alprostadil	Prostaglandin	[M+H-H2O]+	C	380.28, 229.19, 181.16, 156.01, 149.13, 131.00, 15.06
20200124_uo1_apc_plasma_metabolomics_MSMS_POS_17401.CDF	393.2992_12.45	Positive	Bis(2-ethylhexyl) adipate	DEHA		[M+Na]+	C	393.29, 281.17
20200124_uo1_apc_plasma_metabolomics_MSMS_POS_18201.CDF	400.3417_5.79	Positive	Palmitoylcarnitine	Palmitoylcarnitine	Carnitine	[M+H]+	B	400.33, 341.26, 144.09, 85.03
20200124_uo1_apc_plasma_metabolomics_MSMS_POS_22501.CDF	468.3085_5.62	Positive	1-Myristoyl-sn-glycero-3-phosphocholine	LPC(14:0)	LPC	[M+H]+	A	469.30, 468.30, 450.30, 285.24, 258.12, 184.07, 166.06, 163.00, 125.00, 104.11, 86.10
20200124_uo1_apc_plasma_metabolomics_MSMS_POS_23201.CDF	496.3402_6.36	Positive	1-Palmitoyl-sn-glycero-3-phosphocholine	LPC(16:0)	LPC	[M+H]+	A	497.34, 496.34, 478.33, 419.25, 313.27, 258.11, 184.07, 166.06, 163.02, 125.00, 104.11, 86.10, 60.08
20200124_uo1_apc_plasma_metabolomics_MSMS_POS_23301.CDF	508.3752_6.77	Positive	1-(1Z-Octadecenyl)-sn-glycero-3-phosphocholine	LPC(P-18:0)	LPC	[M+H]+	A	508.35, 449.25, 240.13, 184.07, 181.02, 166.06, 136.99, 125.00, 111.07, 104.11, 98.9286.10, 86.10, 83.08
20200124_uo1_apc_plasma_metabolomics_MSMS_POS_23501.CDF	510.3902_7.41	Positive	1-O-Octadecyl-sn-glyceryl-3-phosphorylcholine	LPC(O-18:0)	LPC	[M+H]+	A	510.36, 327.28, 240.09, 184.07, 166.06, 125.00, 104.11, 86.10, 60.08
20200124_uo1_apc_plasma_metabolomics_MSMS_POS_23701.CDF	524.3715_7.15	Positive	1-Stearoyl-2-hydroxy-sn-glycero-3-phosphocholine	LPC(18:0)	LPC	[M+H]+	A	524.37, 506.36, 483.35, 447.29, 341.31, 258.11, 184.07, 125.00, 104.11, 86.10, 60.08
20200124_uo1_apc_plasma_metabolomics_MSMS_POS_24001.CDF	532.3735_7.36	Positive	1-O-Octadecyl-sn-glyceryl-3-phosphorylcholine	LPC(O-18:0)	LPC	[M+H]+	A	532.27, 473.26, 429.24, 398.17, 183.09, 146.98, 104.11, 86.10, 71.09
20200124_uo1_apc_plasma_metabolomics_MSMS_POS_24601.CDF	572.3697_6.61	Positive	1-O-Hexadecyl-2-O-(2E-butenoyl)-sn-glyceryl-3-phosphocholine	Butenoyl PAF	Potent phospholipid activator	[M+Na]+	A	574.38, 573.37, 572.37, 550.30, 514.29, 515.31, 513.29, 367.31, 184.07, 146.98

Supplementary Table S2. List of dysregulated metabolites following exposure to 9.5 Gy of gamma-radiation, 6 months' post irradiation

									Wild Type								APCHI																					
					Overall				Male				Female				Male				Female				Male				Female				Wild Type				APCHI	
mz_rt	name	mode	*p-value*	*FDR*	*Fold Change*	*Log2(FC)*	*p-value*	*FDR* [a]	*Fold Change*	*Log2(FC)*	*p-value*	*FDR*	*Fold Change*	*Log2(FC)*	*p-value*	*FDR*	*Fold Change*	*Log2(FC)*	*p-value*	*FDR*	*Fold Change*	*Log2(FC)*	*p-value*	*FDR*	*Fold Change*	*Log2(FC)*	*p-value*	*FDR*	*Fold Change*	*Log2(FC)*	*p-value*	*FDR*	*Fold Change*	*Log2(FC)*	*p-value*	*FDR*	*Fold Change*	*Log2(FC)*
279.2313_7.37	Pinolenic acid	Positive	6E-06	0	0.64645	-0.62938	0.00012	0.015	0.35362	-1.4997	0.07206	0.48	0.79149	-0.33736	0.01697	0.34	0.71768	-0.47859	0.000020373	0.00090926	0.70793	-0.49832	1.6E-05	0	0.52666	-0.92506	1.8E-06	0	0.56183	-0.83179	0.01619	0.43	0.77883	-0.36061	5.2E-07	0	0.6561	-0.608
261.2205_7.37	Pinolenic acid	Positive	2.9E-05	0	0.66783	-0.58245	9.7E-05	0.015	0.37814	-1.403	0.05009	0.43	0.79492	-0.33111	0.0068	0.26	0.73047	-0.4531	0.000017761	0.00086366	0.72238	-0.46916	6E-06	0	0.55025	-0.86185	2.4E-06	0	0.58443	-0.77489	0.00746	0.28	0.78434	-0.35044	1.9E-07	0	0.67287	-0.57161
277.2155_6.96	Stearidonic acid	Positive	4.1E-05	0	0.59058	-0.75979	0.00193	0.044	0.39001	-1.3584	0.02561	0.35	0.71166	-0.49074	3.3E-05	0.01	0.59891	-0.73959	0.000008075	0.00060692	0.64132	-0.64089	1E-06	0	0.48533	-1.043	1.3E-05	0	0.54974	-0.86319	0.00056	0.07	0.68522	-0.54536	5.8E-08	0	0.60749	-0.71907
283.2631_8.35	(11E)-11-Octadecenoic acid	Positive	0.00038	0.02	0.80846	-0.30674	0.00755	0.084	0.72193	-0.47007	0.04871	0.42	0.8415	-0.24897	0.73244	0.99	0.97129	-0.042023	0.00017908	0.0039158	0.82386	-0.27953	0.0207	0.12	0.86182	-0.21453	0.0059	0.06	0.81985	-0.28656	0.22682	0.88	0.91617	-0.12631	0.00484	0.05	0.86595	-0.20764
190.0857_3.78	3-Indolepropionic acid	Positive	0.00162	0.03	0.32352	-1.6281	0.12304	0.331	0.72912	-0.45577	0.17915	0.62	0.70667	-0.5009	0.01771	0.34	0.65818	-0.60345	0.00088668	0.012044	0.44383	-1.1719	0.00595	0.06	0.66681	-0.58465	0.0043	0.05	0.43638	-1.1964	0.11335	0.77	0.74757	-0.41973	0.0017	0.02	0.54499	-0.8757
277.215_5.83	9(S)-HOTrE	Positive	0.00194	0.04	0.64227	-0.63875	0.00211	0.045	0.53138	-0.91219	0.32716	0.78	0.85972	-0.21806	0.78709	0.99	0.94312	-0.084483	0.0077448	0.057569	0.73086	-0.45234	0.15823	0.4	0.69861	-0.51743	4E-06	0	0.57991	-0.78609	0.65122	0.98	0.90548	-0.14325	0.00374	0.04	0.71337	-0.48727
380.2846_7.99	Prostaglandin E1 ethanolamide	Positive	0.0025	0.04	0.45419	-1.1386	0.03536	0.182	0.24873	-2.0074	0.16524	0.6	0.60361	-0.72831	0.03709	0.4	0.49447	-1.016	0.0049295	0.04097	0.5084	-0.97595	0.00182	0.03	0.35085	-1.5111	0.02816	0.16	0.49317	-1.0198	0.06312	0.66	0.62825	-0.6706	0.00483	0.05	0.53994	-0.88913
327.2322_7.25	(±)10-HDoHE	Positive	0.00358	0.05	0.74977	-0.41548	0.06262	0.24	0.73939	-0.4356	0.25098	0.72	0.8088	-0.30615	0.61912	0.96	0.91531	-0.12767	0.0090562	0.063726	0.77635	-0.36522	0.08201	0.28	0.83387	-0.26211	0.11137	0.33	0.81477	-0.29554	0.52652	0.98	0.88444	-0.17717	0.1231	0.37	0.84637	-0.24064
170.0597_2.01	3-Indolyllactic acid	Positive	0.0123	0.1	0.8294	-0.26987	0.00013	0.015	0.59077	-0.75932	0.64078	0.89	0.96632	-0.04943	0.01192	0.31	0.81968	-0.28687	0.02657	0.13277	0.88893	-0.16986	7.9E-06	0	0.71681	-0.48034	1E-05	0	0.69959	-0.51542	0.02747	0.52	0.8663	-0.20706	2E-06	0	0.78043	-0.35766
307.2631_8.06	5Z,11Z,14Z-Eicosatrienoic acid	Positive	0.02283	0.14	0.80308	-0.31639	0.01397	0.113	0.64882	-0.62411	0.02425	0.34	0.81446	-0.29609	0.55487	0.94	0.91272	-0.13176	0.0010438	0.013549	0.80872	-0.30628	0.02455	0.14	0.79498	-0.33102	0.06566	0.25	0.8204	-0.28561	0.36907	0.94	0.88494	-0.17634	0.04787	0.21	0.85068	-0.23331
188.0706_2.01	Tyr	Positive	0.02295	0.14	0.83964	-0.25215	0.00036	0.021	0.58542	-0.77246	0.98619	1	0.99348	-0.00944	0.00703	0.26	0.80009	-0.32177	0.07978	0.25186	0.90602	-0.14238	7.6E-06	0	0.70616	-0.50193	3.1E-05	0	0.70273	-0.50895	0.03873	0.56	0.86269	-0.21309	7.4E-06	0	0.78087	-0.35685
327.2307_7.71	C18(Plasm)-22:6 PE	Negative	0.02534	0.1	0.82195	-0.28288	0.01315	0.087	0.65595	-0.60834	0.55008	0.85	0.91816	-0.12319	0.20954	0.65	0.84658	-0.24028	0.060613	0.15535	0.86405	-0.21082	0.00659	0.04	0.76193	-0.39227	0.00807	0.05	0.7949	-0.33115	0.24791	0.71	0.89429	-0.16119	0.00743	0.04	0.84133	-0.24926
400.3417_5.79	Palmitoylcarnitine	Positive	0.02567	0.15	1.189	0.24975	0.24073	0.468	1.5045	0.58924	0.00691	0.25	1.2243	0.29191	0.05368	0.46	0.86718	-0.2056	0.00041956	0.0073098	1.2055	0.26968	0.83898	0.92	1.092	0.12696	0.07008	0.25	1.2548	0.32745	0.81719	0.98	1.0086	0.01233	0.09223	0.31	1.1235	0.16801
325.2111_7.86	Maresin 1	Positive	0.02889	0.16	0.87271	-0.19643	0.00203	0.045	0.65603	-0.60816	0.07311	0.49	0.88423	-0.17751	0.03998	0.41	0.87356	-0.19503	0.0044421	0.038287	0.87861	-0.18671	0.00021	0.01	0.77558	-0.36666	0.00016	0.01	0.78645	-0.34657	0.00468	0.26	0.8784	-0.18705	2.4E-06	0	0.83116	-0.26681
369.3519_10.2	Lathosterol	Positive	0.03159	0.17	1.0943	0.13002	0.81437	0.904	1.0096	0.01381	0.36904	0.79	1.0265	0.03774	0.86731	1	1.0057	0.0081513	0.02	0.1112	1.0618	0.086477	0.74786	0.88	1.0077	0.01103	0.27211	0.53	1.0368	0.05218	0.76837	0.98	1.0066	0.00955	0.28629	0.56	1.0211	0.03018
572.3697_6.61	Butenoyl PAF	Positive	0.03419	0.18	0.86773	-0.20469	0.2021	0.427	0.84188	-0.24831	0.3215	0.77	0.94624	-0.07972	0.88064	1	1.0046	0.0065601	0.01878	0.10678	0.90329	-0.14674	0.41353	0.65	0.95009	-0.07386	0.29192	0.55	0.9151	-0.12801	0.99517	1	0.99374	-0.00905	0.3933	0.65	0.95567	-0.06542
144.0805_2.01	1H-Indole-3-ethanol	Positive	0.03528	0.18	0.86188	-0.21444	0.00011	0.015	0.56934	-0.81263	0.87011	0.97	0.98739	-0.01831	0.0096	0.29	0.81324	-0.29824	0.078684	0.24937	0.91712	-0.12481	5.7E-06	0	0.70198	-0.51051	3.9E-05	0	0.69821	-0.51827	0.03329	0.53	0.87013	-0.2007	6.1E-06	0	0.7817	-0.35532
728.557_10.73	C18(Plasm)-18:1 PE	Negative	0.03908	0.13	0.84511	-0.24279	0.04608	0.17	0.75673	-0.40216	0.05736	0.3	0.81308	-0.29853	0.01717	0.24	0.74384	-0.42693	0.0044162	0.021012	0.83122	-0.2667	0.00285	0.02	0.73067	-0.4527	0.0079	0.04	0.83016	-0.26855	0.025	0.3	0.80641	-0.31042	0.00126	0.01	0.81678	-0.29198
159.091_2.01	4-(1-Piperazinyl)-1H-indole1	Positive	0.03971	0.19	0.85225	-0.23066	0.00048	0.025	0.58124	-0.78279	0.78464	0.95	0.97785	-0.03232	0.01884	0.34	0.83354	-0.26268	0.078087	0.24819	0.90706	-0.14073	3.2E-05	0	0.72041	-0.4731	6.3E-05	0	0.70709	-0.50004	0.05048	0.61	0.87799	-0.18773	1.6E-05	0	0.78982	-0.34041
305.2479_7.72	Arachidonic acid	Positive	0.05247	0.23	0.85625	-0.2239	0.14498	0.357	0.85181	-0.23139	0.16793	0.6	0.92396	-0.1141	0.77861	0.99	0.96669	-0.048879	0.014836	0.088852	0.88693	-0.17311	0.238	0.49	0.92277	-0.11596	0.01082	0.09	0.8552	-0.22566	0.30339	0.91	0.94355	-0.08383	0.00877	0.07	0.90073	-0.15084
370.2949_5.1	N-Oleoyl-L-serine	Positive	0.05352	0.23	1.3118	0.3916	0.02859	0.163	2.4883	1.3152	0.01591	0.31	1.3632	0.44705	0.57097	0.94	1.0796	0.11045	0.0028719	0.027908	1.3353	0.4172	0.02927	0.15	1.5532	0.63523	0.0078	0.08	1.6535	0.72554	0.04334	0.58	1.1845	0.24423	0.00085	0.01	1.3963	0.48163
357.2789_5.57	Chenodeoxycholic acid	Positive	0.05912	0.24	1.0671	0.09374	0.5154	0.711	0.87299	-0.19596	0.00201	0.2	1.0984	0.13546	0.02737	0.37	1.1526	0.20491	0.00076818	0.011223	1.0812	0.11266	0.54672	0.75	1.0225	0.03212	0.68469	0.86	0.94944	-0.07485	0.00329	0.19	1.1187	0.16179	0.20643	0.48	1.034	0.04817
850.56_10.13	16:0-22:6 PC	Negative	0.06882	0.19	0.93053	-0.10388	0.21545	0.415	1.1552	0.2082	0.05343	0.3	0.88527	-0.17581	0.58005	0.91	0.96655	-0.04908	0.0069301	0.028606	0.9082	-0.13892	0.43928	0.64	1.0377	0.05343	0.9064	0.96	1.0029	0.00424	0.06072	0.45	0.92576	-0.11129	0.26734	0.5	0.96176	-0.05624
132.0663_0.82	N-Acetyl-L-alanine	Positive	0.07864	0.28	1.0915	0.1263	0.01596	0.123	1.2989	0.37728	0.97995	0.99	1.0021	0.00304	0.88563	1	1.0344	0.048752	0.31836	0.54562	1.0474	0.06685	0.10328	0.32	1.1347	0.18236	0.00863	0.08	1.1488	0.2001	0.98894	1	1.0104	0.015	0.12402	0.37	1.0732	0.10191
508.3752_6.77	C18(Plasm) LPC	Positive	0.0791	0.28	1.1322	0.1791	3.2E-06	0.003	1.7504	0.80767	0.12576	0.55	1.1423	0.1919	0.00013	0.02	1.4122	0.49794	0.027898	0.13668	1.1349	0.18251	3.1E-09	0	1.537	0.62014	0.00765	0.07	1.3014	0.38008	0.00098	0.09	1.2675	0.34196	2.9E-05	0	1.2844	0.36111
288.2527_4.79	Alkamide LE	Positive	0.0867	0.3	1.1033	0.14185	0.92427	0.961	1.0055	0.00796	0.00343	0.24	1.1463	0.19701	0.89866	1	1.005	0.0072592	0.0019079	0.021435	1.1229	0.16729	0.86719	0.93	1.0052	0.00747	0.20347	0.46	1.0502	0.07071	0.03181	0.53	1.0638	0.08929	0.01917	0.12	1.0575	0.08066
162.1125_0.67	L-Carnitine	Positive	0.10908	0.34	0.86387	-0.21112	0.06306	0.24	0.70446	-0.50541	0.16235	0.6	0.88929	-0.16927	0.74565	0.99	0.98993	-0.014609	0.028015	0.13705	0.87565	-0.19157	0.09598	0.31	0.87834	-0.18715	0.30205	0.56	0.89945	-0.15288	0.71926	0.98	0.9639	-0.05304	0.29095	0.57	0.931	-0.10314
393.2992_12.45	Bis(2-ethylhexyl) adipate	Positive	0.12274	0.35	1.0891	0.1231	0.67368	0.823	0.97288	-0.03967	0.03075	0.36	1.0847	0.11723	0.14658	0.64	0.91629	-0.12613	0.012717	0.079674	1.0876	0.12118	0.25773	0.51	0.9463	-0.07964	0.23956	0.5	1.0477	0.06717	0.70597	0.98	0.9794	-0.03004	0.60863	0.81	1.0114	0.01633
120.0807_1.14	N-Ac-Phe	Positive	0.12492	0.36	0.91958	-0.12096	0.32777	0.555	1.0963	0.13267	0.356	0.78	1.0985	0.13548	0.40553	0.87	0.93522	-0.096628	0.95414	0.98001	0.99702	-0.0042997	0.83588	0.92	0.98786	-0.01762	0.43952	0.68	0.96647	-0.0492	0.79525	0.98	1.0116	0.01666	0.75406	0.88	0.98755	-0.01808
121.0283_4.46	5,6-Dihydrothymine	Positive	0.12573	0.36	1.3737	0.45802	0.07011	0.246	1.3153	0.39542	0.04744	0.42	1.42	0.50589	0.35751	0.83	0.92387	-0.11424	0.01573	0.092908	1.3978	0.48319	0.51622	0.73	1.0645	0.09023	0.07865	0.27	1.2894	0.36673	0.68681	0.98	1.0148	0.02118	0.14509	0.4	1.1374	0.18568
365.3182_7.28	7α,24(S)-Dihydroxycholesterol	Positive	0.135	0.37	0.88133	-0.18225	0.23497	0.462	0.84518	-0.24267	0.18552	0.63	0.90214	-0.14858	0.29069	0.78	0.88803	-0.17131	0.039633	0.16788	0.89045	-0.1674	0.10429	0.32	0.87328	-0.19548	0.03677	0.18	0.85844	-0.2202	0.08432	0.72	0.892	-0.16488	0.00659	0.06	0.87668	-0.18988
369.3512_10.89	Lathosterol	Positive	0.18306	0.44	2.2055	1.1411	0.04237	0.198	4.4781	2.1629	0.46412	0.83	0.89704	-0.15675	0.64209	0.97	0.99986	-0.00019629	0.36687	0.58611	1.6004	0.67847	0.03976	0.18	1.9121	0.93513	0.01371	0.1	2.8824	1.5273	0.72519	0.98	0.93021	-0.10438	0.05147	0.22	1.7067	0.77124
380.2556_5.58	Sphingosine-1-Phosphate (d18:1)	Positive	0.33388	0.61	1.0518	0.07292	0.11029	0.314	1.1761	0.23399	0.76016	0.94	1.0074	0.01064	0.2678	0.76	0.92239	-0.11655	0.39835	0.61074	1.033	0.046772	0.92286	0.96	1.0137	0.01956	0.18103	0.43	1.0742	0.1033	0.53198	0.98	0.95181	-0.07126	0.73316	0.87	1.0096	0.01381
468.3085_5.62	14:0 Lyso PC	Positive	0.38149	0.65	1.0443	0.0625	0.43525	0.646	0.93493	-0.09707	0.29998	0.76	1.0714	0.09946	0.0941	0.56	0.88437	-0.17727	0.16098	0.37825	1.0568	0.079713	0.0532	0.22	0.90011	-0.15183	0.46153	0.7	1.0433	0.0611	0.92249	0.99	1.0044	0.00637	0.71334	0.86	1.0226	0.03224
121.0282_5.41	4-OH BnCOOH	Positive	0.48533	0.73	1.0915	0.12628	0.78945	0.892	1.0281	0.04001	0.14903	0.58	1.1082	0.1482	0.37241	0.84	0.94582	-0.08036	0.18576	0.40701	1.101	0.13878	0.60361	0.79	0.97815	-0.03188	0.22699	0.49	1.1184	0.1614	0.62739	0.98	1.0364	0.05158	0.21969	0.5	1.0755	0.10499
329.2086_4.29	Hydrallostane	Positive	0.50553	0.75	0.90931	-0.13716	0.38531	0.605	0.89707	-0.1567	0.38988	0.79	0.79832	-0.32495	0.19246	0.7	0.88364	-0.17847	0.26007	0.48557	0.85084	-0.23304	0.11446	0.34	0.88626	-0.1742	0.1714	0.42	0.69588	-0.52309	0.32023	0.92	0.78965	-0.34071	0.09599	0.32	0.74915	-0.41667
532.3735_7.36	Lyso-PAF C-18	Positive	0.5719	0.79	0.97177	-0.04132	0.23222	0.459	0.87093	-0.19937	0.66848	0.91	1.0334	0.04741	0.06518	0.49	0.85872	-0.21973	0.90322	0.95387	0.99977	-0.00033834	0.02177	0.13	0.85624	-0.22391	0.74529	0.89	0.98265	-0.02525	0.77568	0.98	0.99216	-0.01136	0.65992	0.83	0.98687	-0.01907
370.2712_7.6	N-Arachidonylmaleimide	Positive	0.60533	0.81	0.93265	-0.10058	0.01081	0.1	0.72224	-0.46944	0.35397	0.78	1.0836	0.11579	0.17799	0.68	0.87596	-0.19106	0.72325	0.85214	0.99718	-0.0040714	0.00775	0.07	0.8192	-0.28771	0.12661	0.35	0.88317	-0.17923	0.84562	0.98	0.97052	-0.04317	0.22059	0.5	0.92586	-0.11113
313.0637_0.84	Salicyl glucuronide	Negative	0.67327	0.81	1.014	0.02009	0.2859	0.484	1.067	0.09355	0.48307	0.81	0.96732	-0.04793	0.71704	0.97	0.98557	-0.02097	0.74062	0.84964	0.99236	-0.011059	0.75974	0.87	1.0183	0.02611	0.3741	0.64	1.0268	0.03813	0.39955	0.83	0.97201	-0.04096	0.8319	0.91	0.99786	-0.00308
124.0034_0.69	Taurine	Negative	0.77467	0.88	1.0137	0.0196	0.00339	0.048	1.4261	0.51211	0.24782	0.62	0.85735	-0.22205	0.13896	0.58	1.1894	0.25028	0.43048	0.61188	0.94156	-0.086869	0.02795	0.12	1.2623	0.33606	0.36692	0.63	1.1118	0.15293	0.88537	0.97	1.0147	0.021	0.50633	0.7	1.0615	0.08605
203.0528_0.64	3-Indolyllactic acid	Positive	0.77751	0.91	0.96055	-0.05807	0.24585	0.474	0.87187	-0.19782	0.48852	0.83	0.9424	-0.08558	0.70197	0.98	1.0603	0.084442	0.49566	0.68718	0.95292	-0.069574	0.66261	0.83	0.99423	-0.00835	0.3432	0.6	0.93835	-0.0918	0.90019	0.99	0.99721	-0.00402	0.44393	0.69	0.96913	-0.04524
496.3402_6.36	Lyso-PC(16:0)	Positive	0.84979	0.94	1.0056	0.00801	0.5387	0.728	1.0247	0.03523	0.90362	0.97	0.99938	-0.0009	0.04368	0.42	0.9079	-0.1394	0.83795	0.922	1.0021	0.003042	0.16294	0.4	0.95276	-0.06981	0.36164	0.62	1.0261	0.03716	0.34754	0.93	0.96273	-0.0548	0.82597	0.92	0.99255	-0.01078
524.3715_7.15	18:0 Lyso PC	Positive	0.92457	0.97	1.0077	0.0111	0.12419	0.332	1.1243	0.16901	0.26863	0.73	1.0565	0.07925	0.07124	0.5	0.90994	-0.13616	0.47007	0.66858	1.0296	0.042148	0.90071	0.95	0.99141	-0.01245	0.9728	0.99	1.0087	0.01254	0.43958	0.97	0.95382	-0.06821	0.64863	0.83	0.9797	-0.0296
140.0681_0.64	Spermidic acid	Positive	0.9296	0.97	0.98388	-0.02344	0.24781	0.477	0.80384	-0.31502	0.78601	0.95	0.97268	-0.03996	0.56678	0.94	0.93741	-0.093247	0.77619	0.88677	0.97818	-0.031822	0.1911	0.44	0.86468	-0.20976	0.49508	0.73	0.93535	-0.09642	0.74181	0.98	0.98092	-0.0278	0.4945	0.73	0.95686	-0.06362
510.3902_7.41	Lyso-PAF C-18	Positive	0.94569	0.98	0.99969	-0.00044	0.30442	0.534	1.099	0.13617	0.26933	0.73	1.0563	0.07907	0.06117	0.48	0.87151	-0.19841	0.47893	0.67548	1.0254	0.036196	0.44517	0.68	0.95633	-0.06441	0.95782	0.98	1.0018	0.00261	0.28655	0.91	0.93564	-0.09597	0.42994	0.68	0.96658	-0.04904
319.2636_8.65	ω-3 Arachidonic acid methyl ester	Positive	0.95398	0.98	0.98729	-0.01846	0.00254	0.05	0.50778	-0.97773	0.35681	0.78	1.1445	0.19476	0.73584	0.99	1.1125	0.15379	0.56787	0.74752	1.0578	0.08101	0.17545	0.42	0.88332	-0.17899	0.18355	0.43	0.87775	-0.18812	0.3434	0.93	1.1437	0.19373	0.76178	0.89	1.013	0.0186
329.2481_7.61	cis-5,8,11-Eicosatrienoic acid	Positive	0.9582	0.98	0.98324	-0.02439	0.01721	0.126	0.76231	-0.39156	0.69526	0.92	1.0197	0.02809	0.21492	0.73	0.88094	-0.18289	0.72223	0.85193	1.0008	0.0011206	0.01346	0.09	0.83813	-0.25476	0.36095	0.62	0.9308	-0.10346	0.63412	0.98	0.94542	-0.08098	0.3188	0.59	0.93737	-0.0933
281.2471_7.84	Conjugated linoleic acid	Positive	0.97251	0.99	0.99151	-0.0123	0.0011	0.034	0.76201	-0.39212	0.9586	0.99	1.0001	0.00017	0.24174	0.75	0.93544	-0.096287	0.99908	0.9997	0.99586	-0.005983	0.00239	0.03	0.86477	-0.20961	0.03138	0.17	0.89536	-0.15946	0.32781	0.93	0.96069	-0.05786	0.02382	0.14	0.92853	-0.10698

Supplementary Table S3. List of dysregulated metabolites and lipids upon exposure to 9.5 Gy of gamma radiation, 6 months' post irradiation

								Wild Type								APCHI																					
				Overall				Male				Female				Male				Female				Male				Female				Wild Type				APCHI	
**Mode**	**Name**	***p-value***	***FDR***	***Fold Change***	***Log2(FC)***	***p-value***	***FDR[a]***	***Fold Change***	***Log2(FC)***	***p-value***	***FDR***	***Fold Change***	***Log2(FC)***	***p-value***	***FDR***	***Fold Change***	***Log2(FC)***	***p-value***	***FDR***	***Fold Change***	***Log2(FC)***	***p-value***	***FDR***	***Fold Change***	***Log2(FC)***	***p-value***	***FDR***	***Fold Change***	***Log2(FC)***	***p-value***	***FDR***	***Fold Change***	***Log2(FC)***	***p-value***	***FDR***	***Fold Change***	***Log2(FC)***
Metabolomics	Phosphorylcholine	0.14498	0.73	0.89963	-0.1526	0.02456	0.94	0.71276	-0.4885	0.90032	1	0.98504	-0.0217	0.39624	0.98	0.89198	-0.1649	0.62441	0.99	1.1016	0.1396	0.01521	0.65	0.78912	-0.3417	0.78527	1	1.0446	0.063	0.07037	0.81	0.82543	-0.2768	0.90367	1	0.98278	-0.0251
Metabolomics	Citric Acid	0.01858	0.3	1.4932	0.57841	0.03004	0.94	1.7505	0.8078	0.30341	0.93	1.5786	0.65867	0.30363	0.98	1.5724	0.65297	0.79993	0.99	0.90841	-0.1386	0.0132	0.65	1.7591	0.81481	0.62737	1	1.135	0.18271	0.01747	0.68	1.7626	0.81767	0.34738	1	1.1839	0.2435
Metabolomics	Glycerophosphocholine	0.07828	0.73	0.77966	-0.3591	0.03602	0.94	0.57587	-0.7962	0.60849	0.96	0.89831	-0.1547	0.3571	0.98	0.75023	-0.4146	0.98612	0.99	1.0418	0.05905	0.02171	0.65	0.64804	-0.6258	0.71201	1	0.97116	-0.0422	0.06672	0.81	0.70912	-0.4959	0.51723	1	0.85842	-0.2203
Metabolomics	Xanthylic Acid	0.02209	0.32	1.3382	0.42026	0.03997	0.94	1.5047	0.5895	0.62303	0.96	1.3874	0.47241	0.62187	0.98	1.1413	0.19067	0.26394	0.99	1.3465	0.42925	0.07213	0.73	1.2652	0.33932	0.2687	1	1.3159	0.39604	0.03911	0.68	1.4884	0.5738	0.28466	1	1.2109	0.27614
Metabolomics	Hypoxanthine	0.40843	0.85	1.0732	0.10194	0.04113	0.94	1.5425	0.62529	0.96841	1	0.85225	-0.2307	0.10149	0.98	0.707	-0.5002	0.57552	0.99	1.3144	0.39443	0.55501	0.92	1.0517	0.07275	0.72709	1	1.0605	0.08475	0.11461	0.85	1.2319	0.3009	0.7177	1	0.9302	-0.1044
Metabolomics	4-Aminobenzoic Acid	0.27074	0.73	1.2314	0.30028	0.04436	0.94	1.5912	0.6701	0.15419	0.93	0.49607	-1.0114	0.16975	0.98	1.6781	0.74682	0.29507	0.99	0.81426	-0.2964	0.0126	0.65	1.6362	0.71035	0.16705	1	0.74086	-0.4327	0.39468	0.85	1.2854	0.36218	0.50275	1	1.1806	0.23956
Metabolomics	N-Acetylornithine	1E-04	0.01	0.61953	-0.6908	0.04687	0.94	0.70607	-0.5021	0.00506	0.48	0.32702	-1.6126	0.70591	1	0.9337	-0.099	0.03487	0.99	0.62912	-0.6686	0.06349	0.68	0.80001	-0.3219	0.00044	0.04	0.50278	-0.992	0.00031	0.03	0.50712	-0.9796	0.08401	1	0.74961	-0.4158
Metabolomics	Glutathione Reduced	0.23477	0.73	1.062	0.08676	0.04893	0.94	1.4936	0.57875	0.40037	0.93	1.1687	0.2249	0.40494	0.98	0.7885	-0.3428	0.55785	0.99	1.0685	0.09552	0.51756	0.92	1.0306	0.0435	0.26118	1	1.1049	0.14397	0.06188	0.81	1.2756	0.35121	0.72712	1	0.90817	-0.139
Metabolomics	Methylthioadenosine	0.13261	0.73	0.93995	-0.0893	0.05104	0.94	0.57384	-0.8013	0.49576	0.94	1.3829	0.46771	0.24394	0.98	0.94601	-0.0801	0.30585	0.99	1.3697	0.45383	0.01895	0.65	0.72423	-0.4655	0.23966	1	1.3274	0.40857	0.12583	0.85	0.78988	-0.3403	0.59126	1	1.0937	0.12924
Metabolomics	Arginine	6.6E-05	0.01	0.61579	-0.6995	0.05125	0.94	0.715	-0.484	0.00424	0.48	0.32615	-1.6164	0.54359	0.98	0.89691	-0.157	0.04151	0.99	0.63944	-0.6451	0.04438	0.65	0.79002	-0.34	0.00046	0.04	0.50761	-0.9782	0.0003	0.03	0.50443	-0.9873	0.06296	1	0.74272	-0.4291
Metabolomics	Uracil	0.10109	0.73	1.1092	0.14953	0.07577	0.94	1.2571	0.33014	0.11095	0.93	1.2459	0.31716	0.10081	0.98	1.197	0.25943	0.5228	0.99	0.8696	-0.2016	0.01864	0.65	1.2174	0.28378	0.97778	1	0.98754	-0.0181	0.07218	0.81	1.1864	0.24662	0.60853	1	1.0324	0.04604
Metabolomics	Dopamine	0.5142	0.89	1.1844	0.2441	0.14861	0.94	1.3031	0.3819	0.16153	0.93	1.4517	0.53771	0.34705	0.98	0.9285	-0.107	0.81016	0.99	1.0614	0.08598	0.75664	0.95	1.1859	0.24602	0.39613	1	1.1817	0.2409	0.03807	0.68	1.3506	0.43362	0.46411	1	0.99623	-0.0054
Metabolomics	Proline	0.009	0.25	0.80794	-0.3077	0.17569	0.94	0.82984	-0.2691	0.40051	0.93	0.81513	-0.2949	0.17996	0.98	0.8199	-0.2865	0.62146	0.99	0.95392	-0.0681	0.04214	0.65	0.81873	-0.2885	0.20236	1	0.83864	-0.2539	0.03025	0.68	0.74313	-0.4283	0.16029	1	0.88471	-0.1767
Metabolomics	Glycine	0.01563	0.27	0.71709	-0.4798	0.20866	0.94	0.80185	-0.3186	0.71903	0.97	0.90142	-0.1497	0.14454	0.98	0.8518	-0.2314	0.28864	0.99	0.84102	-0.2498	0.04398	0.65	0.81708	-0.2915	0.17244	1	0.77251	-0.3724	0.06126	0.81	0.63568	-0.6536	0.107	1	0.84549	-0.2421
Metabolomics	Xanthurenic Acid	0.00713	0.25	0.72704	-0.4599	0.2105	0.94	0.69168	-0.5318	0.20239	0.93	0.66414	-0.5904	0.06149	0.98	0.72435	-0.4652	0.89646	0.99	0.99431	-0.0082	0.02841	0.65	0.70492	-0.5045	0.19666	1	0.77992	-0.3586	0.02697	0.68	0.63368	-0.6582	0.14256	1	0.84674	-0.24
Metabolomics	Indoleacrylic Acid	0.00751	0.25	0.7711	-0.375	0.23268	0.94	0.77796	-0.3622	0.17543	0.93	0.69214	-0.5309	0.04996	0.98	0.73696	-0.4404	0.97952	0.99	1.0149	0.02129	0.02597	0.65	0.75765	-0.4004	0.22361	1	0.82081	-0.2849	0.02396	0.68	0.6906	-0.5341	0.16772	1	0.86797	-0.2043
Metabolomics	Tryptophan	0.01077	0.25	0.72132	-0.4713	0.29173	0.94	0.739	-0.4364	0.17647	0.93	0.64009	-0.6437	0.04829	0.98	0.67167	-0.5742	0.92234	0.99	1.0226	0.03226	0.03407	0.65	0.70386	-0.5066	0.26536	1	0.78811	-0.3435	0.03086	0.68	0.627	-0.6735	0.19268	1	0.8376	-0.2557
Metabolomics	Indole	0.00809	0.25	0.74813	-0.4186	0.31663	0.94	0.79754	-0.3264	0.19559	0.93	0.6773	-0.5621	0.05173	0.98	0.70319	-0.508	0.9068	0.99	0.99909	-0.0013	0.03456	0.65	0.74688	-0.4211	0.19151	1	0.78937	-0.3412	0.0339	0.68	0.67273	-0.5719	0.13008	1	0.83963	-0.2522
Metabolomics	N-Acetylornithine	0.01357	0.26	0.7598	-0.3963	0.3354	0.94	0.71061	-0.4929	0.03988	0.93	0.48755	-1.0364	0.93292	1	1.1541	0.20683	0.1201	0.99	0.7667	-0.3833	0.4014	0.89	0.88615	-0.1744	0.00763	0.49	0.6474	-0.6273	0.02433	0.68	0.60196	-0.7323	0.2633	1	0.93977	-0.0896
Metabolomics	4-Aminobenzoic Acid	0.01052	0.25	0.86395	-0.211	0.87863	0.988	1.0263	0.03746	0.05787	0.93	0.74428	-0.4261	0.01307	0.98	0.80077	-0.3205	0.46874	0.99	0.90641	-0.1418	0.14964	0.89	0.91476	-0.1285	0.04121	1	0.80626	-0.3107	0.12972	0.85	0.87694	-0.1894	0.03336	1	0.85008	-0.2343
Lipidomics	LPC(22:0)	0.30446	0.49	0.87237	-0.197	0.00145	0.107	0.72071	-0.4725	0.45007	0.68	1.0947	0.13056	0.42195	0.86	0.8101	-0.3038	0.52384	0.74	0.92206	-0.1171	0.01002	0.07	0.75561	-0.4043	0.81024	0.89	0.98395	-0.0233	0.54777	0.8	0.89582	-0.1587	0.41847	0.68	0.84944	-0.2354
Lipidomics	PE(O-18:0/22:4)	0.3515	0.54	0.89624	-0.158	0.00264	0.107	0.59151	-0.7575	0.47678	0.71	1.0853	0.11806	0.09528	0.61	1.3327	0.41435	0.16944	0.47	0.82103	-0.2845	0.34357	0.48	0.84535	-0.2424	0.41108	0.59	0.90842	-0.1386	0.0479	0.18	0.7574	-0.4009	0.48698	0.7	1.0797	0.1107
Lipidomics	PC(16:0/18:2)	0.01648	0.06	0.78062	-0.3573	0.0028	0.107	0.69841	-0.5179	0.06896	0.28	0.74912	-0.4167	0.30025	0.82	0.78408	-0.3509	0.15371	0.46	0.76887	-0.3792	0.00513	0.05	0.73755	-0.4392	0.01466	0.07	0.7446	-0.4255	0.03389	0.16	0.78274	-0.3534	0.1606	0.52	0.77825	-0.3617
Lipidomics	LPC(20:0)	0.90655	0.95	0.88683	-0.1733	0.00316	0.107	0.72191	-0.4701	0.78224	0.91	0.9428	-0.085	0.93777	0.98	0.85584	-0.2246	0.29553	0.58	0.70037	-0.5138	0.14414	0.28	0.778	-0.3622	0.25594	0.43	0.79571	-0.3297	0.70843	0.86	0.95667	-0.0639	0.64436	0.82	0.81703	-0.2915
Lipidomics	PC(17:0/18:2)	0.00039	0.01	0.78459	-0.35	0.00325	0.107	0.7348	-0.4446	0.12453	0.38	0.74136	-0.4318	0.19133	0.73	0.84291	-0.2466	0.01846	0.36	0.77587	-0.3661	0.00168	0.04	0.78214	-0.3545	0.00339	0.04	0.73332	-0.4475	0.00247	0.07	0.75922	-0.3974	0.03587	0.4	0.814	-0.2969
Lipidomics	PC(17:0/22:6)	0.01	0.05	0.85124	-0.2324	0.00425	0.107	0.7662	-0.3842	0.30093	0.56	0.84319	-0.2461	0.39905	0.86	0.90137	-0.1498	0.11471	0.43	0.82788	-0.2725	0.00603	0.05	0.82339	-0.2804	0.05215	0.14	0.83803	-0.2549	0.04586	0.18	0.83497	-0.2602	0.1136	0.49	0.8678	-0.2046
Lipidomics	PI 16_0-20_1	0.00027	0.01	0.78557	-0.3482	0.00454	0.107	0.73527	-0.4436	0.00193	0.11	0.62605	-0.6757	0.18336	0.73	0.8356	-0.2591	0.22837	0.51	0.83499	-0.2602	0.00209	0.04	0.78129	-0.3561	0.00361	0.04	0.74278	-0.429	0.00057	0.03	0.7414	-0.4317	0.08967	0.45	0.83532	-0.2596
Lipidomics	PE(O-18:0/18:1)	0.01567	0.06	0.77597	-0.3659	0.00534	0.107	0.62561	-0.6767	0.3754	0.63	0.84319	-0.2461	0.79048	0.92	0.89831	-0.1547	0.10103	0.42	0.7326	-0.4489	0.0238	0.11	0.73703	-0.4402	0.03435	0.11	0.76185	-0.3924	0.02766	0.14	0.73622	-0.4418	0.20647	0.53	0.82461	-0.2782
Lipidomics	PC(18:0/18:3)	2.9E-06	0	0.61757	-0.6953	0.00563	0.107	0.56849	-0.8148	0.00372	0.13	0.55583	-0.8473	0.05112	0.5	0.69939	-0.5158	0.00368	0.19	0.58537	-0.7726	0.00054	0.04	0.6264	-0.6749	5E-05	0.01	0.53978	-0.8896	0.00014	0.03	0.59074	-0.7594	0.00353	0.16	0.65221	-0.6166
Lipidomics	PE(18:2/16:1)	0.00319	0.03	0.74147	-0.4315	0.00678	0.107	0.66429	-0.5901	0.02754	0.2	0.62156	-0.686	0.30507	0.82	0.81931	-0.2875	0.14568	0.46	0.76825	-0.3804	0.00806	0.07	0.73389	-0.4464	0.00611	0.04	0.66824	-0.5816	0.00351	0.07	0.69772	-0.5193	0.13502	0.49	0.7986	-0.3245
Lipidomics	PC(20:0/22:6)	0.10882	0.24	0.75117	-0.4128	0.0068	0.107	0.64557	-0.6314	0.89268	0.96	0.94225	-0.0858	0.20592	0.73	0.69821	-0.5183	0.0941	0.42	0.63191	-0.6622	0.00419	0.05	0.66999	-0.5778	0.08764	0.2	0.74331	-0.428	0.52422	0.78	0.82626	-0.2753	0.12451	0.49	0.67628	-0.5643
Lipidomics	PC(16:0/22:5)	0.22414	0.41	0.81062	-0.3029	0.00686	0.107	0.70232	-0.5098	0.68251	0.88	0.92431	-0.1136	0.17915	0.73	0.71038	-0.4933	0.40838	0.66	0.8028	-0.3169	0.00558	0.05	0.71045	-0.4932	0.37957	0.56	0.86523	-0.2088	0.69421	0.85	0.87925	-0.1857	0.2066	0.53	0.74296	-0.4286
Lipidomics	PC(16:0/22:6)	0.06477	0.17	0.8359	-0.2586	0.00689	0.107	0.73346	-0.4472	0.61104	0.83	0.92701	-0.1093	0.17979	0.73	0.79862	-0.3244	0.17644	0.47	0.80509	-0.3128	0.00359	0.05	0.76463	-0.3872	0.1243	0.25	0.85394	-0.2278	0.32729	0.6	0.86975	-0.2013	0.11628	0.49	0.8012	-0.3198
Lipidomics	LPC(18:2)	0.13568	0.28	0.9186	-0.1225	0.00692	0.107	0.80572	-0.3117	0.80658	0.91	0.96052	-0.0581	0.70364	0.91	0.9424	-0.0856	0.78565	0.9	0.98934	-0.0155	0.02318	0.11	0.8622	-0.2139	0.48842	0.64	0.95369	-0.0684	0.06095	0.21	0.87854	-0.1868	0.70921	0.86	0.96229	-0.0555
Lipidomics	PC(16:0/18:3)	0.00038	0.01	0.66756	-0.583	0.00719	0.107	0.62139	-0.6864	0.01606	0.16	0.63893	-0.6463	0.11054	0.61	0.67926	-0.558	0.00954	0.32	0.57258	-0.8044	0.00171	0.04	0.65033	-0.6208	0.00019	0.01	0.58437	-0.775	0.00819	0.08	0.69386	-0.5273	0.01461	0.31	0.63798	-0.6484
Lipidomics	PA 18_1-20_3	0.00452	0.03	0.82431	-0.2787	0.00769	0.107	0.74857	-0.4178	0.03204	0.21	0.73504	-0.4441	0.1903	0.73	0.85084	-0.2331	0.59077	0.77	0.89933	-0.1531	0.00328	0.05	0.79531	-0.3304	0.07018	0.17	0.82033	-0.2857	0.00361	0.07	0.7817	-0.3553	0.24283	0.55	0.87238	-0.197
Lipidomics	PE(P-18:0/22:6)	0.00069	0.01	0.76321	-0.3899	0.00868	0.107	0.68731	-0.541	0.3031	0.56	0.82319	-0.2807	0.20195	0.73	0.79154	-0.3373	0.0957	0.42	0.78123	-0.3562	0.00338	0.05	0.73153	-0.451	0.02607	0.09	0.77571	-0.3664	0.00459	0.08	0.74171	-0.4311	0.04755	0.41	0.78699	-0.3456
Lipidomics	PC(18:2/20:5)	0.00657	0.04	0.66285	-0.5932	0.00899	0.107	0.62919	-0.6684	0.06938	0.28	0.59012	-0.7609	0.17938	0.73	0.67479	-0.5675	0.10064	0.42	0.54274	-0.8817	0.00384	0.05	0.65161	-0.6179	0.0091	0.06	0.55187	-0.8576	0.03258	0.15	0.69797	-0.5188	0.07484	0.44	0.62485	-0.6784
Lipidomics	PC(20:0/18:2)	0.26027	0.46	0.80566	-0.3118	0.009	0.107	0.73973	-0.4349	0.02508	0.19	0.63643	-0.6519	0.49904	0.89	0.78712	-0.3454	0.07932	0.41	0.5312	-0.9127	0.05002	0.17	0.76599	-0.3846	0.0048	0.04	0.55832	-0.8408	0.6563	0.85	0.88667	-0.1735	0.27806	0.59	0.71497	-0.4841
Lipidomics	PC(15:0/18:1)	0.1421	0.29	0.84313	-0.2462	0.00905	0.107	0.73517	-0.4439	0.28182	0.54	0.78071	-0.3571	0.35634	0.86	0.83061	-0.2678	0.54066	0.74	0.88022	-0.1841	0.01419	0.09	0.78059	-0.3574	0.21834	0.39	0.84039	-0.2509	0.2514	0.51	0.83669	-0.2572	0.36192	0.65	0.85023	-0.2341
Lipidomics	PC(20:0/20:4)	0.27385	0.46	0.79384	-0.3331	0.00911	0.107	0.69041	-0.5345	0.30685	0.57	0.81497	-0.2952	0.56195	0.89	0.78065	-0.3572	0.08533	0.41	0.5776	-0.7919	0.03894	0.14	0.73108	-0.4519	0.02444	0.09	0.65816	-0.6035	0.71263	0.86	0.8634	-0.2119	0.27676	0.59	0.72022	-0.4735
Lipidomics	DAG(18:1/20:5)	9.7E-06	0	0.42857	-1.2224	0.00922	0.107	0.3838	-1.3816	0.02123	0.19	0.3241	-1.6255	0.00848	0.5	0.40735	-1.2957	0.11794	0.43	0.54506	-0.8755	0.00027	0.04	0.40594	-1.3007	0.00346	0.04	0.41428	-1.2713	0.00075	0.03	0.407	-1.2969	0.0042	0.16	0.46111	-1.1168
Lipidomics	DAG(18:2/20:4)	6.2E-06	0	0.41895	-1.2552	0.01157	0.125	0.43496	-1.201	0.02883	0.2	0.25338	-1.9806	0.05098	0.5	0.46841	-1.0942	0.04525	0.4	0.59459	-0.75	0.00108	0.04	0.45861	-1.1247	0.00205	0.03	0.34225	-1.5469	0.00028	0.03	0.36508	-1.4537	0.0058	0.16	0.51641	-0.9534
Lipidomics	PC(16:0/20:4)	0.0471	0.14	0.80118	-0.3198	0.012	0.125	0.66528	-0.588	0.21881	0.5	0.84008	-0.2514	0.17704	0.73	0.74371	-0.4272	0.60269	0.78	0.92165	-0.1177	0.00482	0.05	0.70184	-0.5108	0.24913	0.43	0.89425	-0.1613	0.12489	0.34	0.79159	-0.3372	0.2209	0.55	0.81141	-0.3015
Lipidomics	LPA 18_2	0.14915	0.3	0.84133	-0.2493	0.01217	0.125	0.71063	-0.4928	0.05137	0.26	0.58004	-0.7858	0.95821	0.98	0.98723	-0.0185	0.83564	0.92	1.0193	0.02759	0.1014	0.23	0.82616	-0.2755	0.22223	0.4	0.78821	-0.3434	0.01809	0.12	0.72655	-0.4609	0.85082	0.95	1.001	0.00138
Lipidomics	PC(18:2/22:5)	0.45057	0.64	0.86024	-0.2172	0.01324	0.126	0.78885	-0.3422	0.91084	0.96	0.84971	-0.235	0.54914	0.89	0.82217	-0.2825	0.08482	0.41	0.73221	-0.4497	0.05672	0.18	0.80083	-0.3204	0.3375	0.52	0.79878	-0.3241	0.87172	0.97	0.94027	-0.0888	0.19739	0.53	0.78824	-0.3433
Lipidomics	PE(O-18:0/18:2)	0.01425	0.06	0.77074	-0.3757	0.01365	0.126	0.65974	-0.6	0.37532	0.63	0.8443	-0.2442	0.47043	0.86	0.83932	-0.2527	0.1126	0.43	0.72914	-0.4557	0.01635	0.1	0.73618	-0.4419	0.03732	0.11	0.74694	-0.4209	0.02639	0.14	0.75096	-0.4132	0.16605	0.52	0.79427	-0.3323
Lipidomics	LPC(24:0)	0.30065	0.49	0.91188	-0.1331	0.01392	0.126	0.79054	-0.3391	0.80988	0.91	1.0205	0.02928	0.79314	0.92	0.92645	-0.1102	0.17742	0.47	0.86289	-0.2128	0.0653	0.18	0.84614	-0.241	0.30057	0.49	0.92528	-0.112	0.59136	0.81	0.92382	-0.1143	0.38447	0.67	0.90016	-0.1518
Lipidomics	PC(16:0/16:0)	0.00177	0.02	0.78736	-0.3449	0.01432	0.126	0.74605	-0.4227	0.11661	0.36	0.76315	-0.39	0.14982	0.73	0.77598	-0.3659	0.07771	0.41	0.80085	-0.3204	0.00395	0.05	0.75646	-0.4027	0.00901	0.06	0.77262	-0.3722	0.01497	0.11	0.78838	-0.343	0.04719	0.41	0.78631	-0.3468
Lipidomics	PC(17:0/20:4)	0.00737	0.04	0.82899	-0.2706	0.01517	0.129	0.73318	-0.4478	0.1771	0.48	0.79172	-0.3369	0.06115	0.56	0.77966	-0.3591	0.85291	0.93	1.017	0.02429	0.00151	0.04	0.75508	-0.4053	0.2906	0.47	0.90584	-0.1427	0.01642	0.11	0.78399	-0.3511	0.19347	0.53	0.87896	-0.1861
Lipidomics	PC(18:2/18:2)	0.00646	0.04	0.75493	-0.4056	0.01605	0.133	0.71886	-0.4762	0.01111	0.16	0.57347	-0.8022	0.59415	0.89	0.86735	-0.2053	0.03434	0.4	0.6832	-0.5496	0.02923	0.12	0.78206	-0.3547	0.00074	0.01	0.59858	-0.7404	0.00493	0.08	0.72099	-0.472	0.1722	0.52	0.79663	-0.328
Lipidomics	PC(16:0/20:5)	0.00231	0.02	0.66963	-0.5786	0.01692	0.134	0.62303	-0.6826	0.07688	0.3	0.66364	-0.5915	0.07288	0.58	0.61986	-0.69	0.08931	0.42	0.64442	-0.6339	0.00222	0.04	0.6263	-0.6751	0.00851	0.06	0.64009	-0.6436	0.03069	0.15	0.70704	-0.5001	0.03187	0.4	0.62895	-0.669
Lipidomics	PC(18:0/18:2)	3.2E-05	0	0.79739	-0.3267	0.01725	0.134	0.76555	-0.3854	0.06507	0.28	0.79541	-0.3302	0.09854	0.61	0.83976	-0.252	0.01537	0.36	0.79258	-0.3354	0.00246	0.04	0.79687	-0.3276	0.00124	0.02	0.77236	-0.3727	0.00134	0.05	0.77847	-0.3613	0.00863	0.22	0.8181	-0.2897
Lipidomics	PE(18:2/18:2)	0.00623	0.04	0.69336	-0.5283	0.01831	0.135	0.62527	-0.6774	0.07875	0.3	0.62772	-0.6718	0.91282	0.97	0.8396	-0.2522	0.00647	0.28	0.53398	-0.9051	0.08452	0.2	0.71452	-0.485	0.00063	0.01	0.54736	-0.8695	0.01318	0.1	0.67514	-0.5668	0.1058	0.49	0.71576	-0.4824
Lipidomics	PC(18:2/20:1)	0.3871	0.58	0.84936	-0.2356	0.01839	0.135	0.78055	-0.3574	0.02132	0.19	0.58978	-0.7618	0.75578	0.91	0.85124	-0.2324	0.17107	0.47	0.6318	-0.6625	0.15499	0.29	0.81551	-0.2942	0.00861	0.06	0.60815	-0.7175	0.68251	0.85	0.90806	-0.1391	0.4339	0.68	0.78455	-0.3501
Lipidomics	PI 20_0-20_3	0.00126	0.02	0.65144	-0.6183	0.01999	0.139	0.58785	-0.7665	0.01283	0.16	0.42932	-1.2199	0.11259	0.61	0.70128	-0.5119	0.2476	0.52	0.6809	-0.5545	0.00392	0.05	0.63801	-0.6483	0.00939	0.06	0.58104	-0.7833	0.00709	0.08	0.61463	-0.7022	0.08719	0.45	0.69297	-0.5291
Lipidomics	PE(P-18:1/22:6)	0.56956	0.73	0.91786	-0.1237	0.02015	0.139	0.72332	-0.4673	0.27619	0.54	1.1492	0.20069	0.53168	0.89	0.86747	-0.2051	0.48193	0.71	1.0916	0.12641	0.02532	0.11	0.78272	-0.3534	0.25073	0.43	1.0984	0.13544	0.44632	0.73	0.88323	-0.1791	0.96782	0.99	0.95436	-0.0674
Lipidomics	PG 16_0-18_1_2	0.0046	0.03	0.74281	-0.4289	0.02064	0.139	0.7163	-0.4814	0.00595	0.13	0.62698	-0.6735	0.02639	0.5	0.69568	-0.5235	0.17378	0.47	0.73347	-0.4472	0.00111	0.04	0.71187	-0.4903	0.00757	0.05	0.67669	-0.5634	0.03212	0.15	0.77305	-0.3714	0.05595	0.41	0.70974	-0.4946
Lipidomics	LPC(22:1)	0.84628	0.93	0.91854	-0.1226	0.02227	0.143	0.80443	-0.314	0.90354	0.96	0.99033	-0.014	0.76209	0.91	0.88214	-0.1809	0.22096	0.51	0.72065	-0.4726	0.12203	0.25	0.83488	-0.2604	0.224	0.4	0.82472	-0.278	0.59781	0.81	1.011	0.01571	0.50165	0.71	0.83173	-0.2658
Lipidomics	LPC(18:0)	0.65251	0.79	0.99201	-0.0116	0.02239	0.143	0.82334	-0.2805	0.02685	0.2	1.307	0.38625	0.88345	0.95	0.99148	-0.0124	0.71742	0.85	1.0594	0.0832	0.09739	0.22	0.89113	-0.1663	0.08021	0.19	1.1553	0.20832	0.43451	0.72	0.95668	-0.0639	0.91282	0.97	1.0258	0.03681
Lipidomics	TAG52:2-FA16:0	0.00349	0.03	0.48272	-1.0507	0.02324	0.144	0.48568	-1.0419	0.02147	0.19	0.30044	-1.7348	0.45807	0.86	0.64649	-0.6293	0.0511	0.41	0.23027	-2.1186	0.02221	0.11	0.5506	-0.8609	0.0023	0.03	0.26427	-1.9199	0.02057	0.13	0.51895	-0.9463	0.0696	0.44	0.45141	-1.1475
Lipidomics	PC(18:0/20:1)	0.13536	0.28	0.82783	-0.2726	0.02434	0.144	0.75824	-0.3993	0.21054	0.49	0.81441	-0.2962	0.39714	0.86	0.83066	-0.2677	0.08483	0.41	0.65302	-0.6148	0.0272	0.12	0.79075	-0.3387	0.02423	0.09	0.71735	-0.4793	0.54973	0.8	0.89268	-0.1638	0.1575	0.52	0.76354	-0.3892
Lipidomics	PE(P-18:2/18:2)	0.00184	0.02	0.68573	-0.5443	0.02467	0.144	0.59789	-0.7421	0.06067	0.28	0.61086	-0.7111	0.06552	0.58	0.65557	-0.6092	0.75341	0.87	0.88461	-0.1769	0.00362	0.05	0.62951	-0.6677	0.10437	0.23	0.73001	-0.454	0.00514	0.08	0.6372	-0.6502	0.10114	0.49	0.74756	-0.4197
Lipidomics	DAG(18:2/20:5)	3E-06	0	0.45217	-1.1451	0.02488	0.144	0.40101	-1.3183	0.01318	0.16	0.36306	-1.4617	0.01293	0.5	0.49101	-1.0262	0.01764	0.36	0.56397	-0.8263	0.00135	0.04	0.44798	-1.1585	0.00035	0.01	0.41913	-1.2545	0.00077	0.03	0.41253	-1.2774	0.00052	0.11	0.52162	-0.9389
Lipidomics	PC(14:0/18:2)	0.23033	0.41	0.83187	-0.2656	0.02557	0.144	0.74919	-0.4166	0.13945	0.41	0.72898	-0.4561	0.73853	0.91	0.85633	-0.2238	0.09591	0.42	0.66617	-0.586	0.10425	0.23	0.79765	-0.3262	0.01454	0.07	0.68285	-0.5504	0.5269	0.78	0.87024	-0.2005	0.30479	0.61	0.79016	-0.3398
Lipidomics	LPC(24:1)	0.53992	0.71	0.92808	-0.1077	0.02716	0.144	0.81428	-0.2964	0.48919	0.72	0.93138	-0.1026	0.97062	0.98	0.94523	-0.0813	0.30324	0.58	0.84927	-0.2357	0.14701	0.28	0.86654	-0.2067	0.22101	0.4	0.89025	-0.1677	0.77736	0.9	0.94873	-0.0759	0.58782	0.78	0.90826	-0.1388
Lipidomics	PE(O-18:0/20:4)	0.12337	0.26	0.86333	-0.212	0.02777	0.144	0.69624	-0.5224	0.99147	1	0.96492	-0.0515	0.75482	0.91	1.004	0.00574	0.45855	0.7	0.89829	-0.1548	0.1159	0.24	0.82085	-0.2848	0.35603	0.54	0.88597	-0.1747	0.05276	0.19	0.78764	-0.3444	0.82711	0.93	0.95731	-0.0629
Lipidomics	TAG54:5-FA20:4	0.00089	0.01	0.51652	-0.9531	0.02861	0.144	0.50943	-0.973	0.00549	0.13	0.29669	-1.753	0.41975	0.86	0.65856	-0.6026	0.02516	0.38	0.30202	-1.7273	0.02168	0.11	0.57055	-0.8096	0.00029	0.01	0.30684	-1.7045	0.00835	0.08	0.52826	-0.9207	0.04246	0.41	0.50492	-0.9859
Lipidomics	PG 16_1-18_2_2	0.00149	0.02	0.8232	-0.2807	0.02873	0.144	0.79637	-0.3285	0.04839	0.26	0.76757	-0.3816	0.00723	0.5	0.78179	-0.3551	0.35285	0.61	0.91773	-0.1239	0.00052	0.04	0.7866	-0.3463	0.02421	0.09	0.82145	-0.2838	0.00692	0.08	0.81176	-0.3009	0.06879	0.44	0.83568	-0.259
Lipidomics	PA 18_0-18_1	0.02729	0.1	0.8591	-0.2191	0.02894	0.144	0.80002	-0.3219	0.0397	0.23	0.78084	-0.3569	0.24798	0.76	0.85012	-0.2343	0.7964	0.9	0.93316	-0.0998	0.01897	0.11	0.82768	-0.2729	0.14005	0.28	0.85765	-0.2215	0.02047	0.13	0.83435	-0.2613	0.35329	0.65	0.88705	-0.1729
Lipidomics	PC(18:2/20:3)	0.62186	0.78	0.87679	-0.1897	0.0303	0.144	0.76751	-0.3817	0.40945	0.66	0.84492	-0.2431	0.58158	0.89	0.81546	-0.2943	0.55268	0.74	0.82053	-0.2854	0.08913	0.21	0.79011	-0.3399	0.30406	0.49	0.83163	-0.266	0.96994	0.99	0.93485	-0.0972	0.55373	0.75	0.81704	-0.2915
Lipidomics	LPC(18:3)	0.00166	0.02	0.79963	-0.3226	0.03085	0.144	0.80146	-0.3193	0.09037	0.32	0.72227	-0.4694	0.40821	0.86	0.87601	-0.191	0.00292	0.19	0.69732	-0.5201	0.02328	0.11	0.83174	-0.2658	0.00052	0.01	0.68091	-0.5545	0.01271	0.1	0.79937	-0.3231	0.04049	0.41	0.79992	-0.3221
Lipidomics	PC(15:0/18:2)	0.06543	0.17	0.85443	-0.227	0.03146	0.144	0.79706	-0.3272	0.10439	0.34	0.72501	-0.4639	0.87708	0.95	0.95512	-0.0662	0.03762	0.4	0.73613	-0.442	0.10676	0.23	0.86601	-0.2076	0.0038	0.04	0.71535	-0.4833	0.1453	0.36	0.84586	-0.2415	0.23248	0.55	0.86415	-0.2106
Lipidomics	PC(18:0/20:0)	0.0106	0.05	0.84364	-0.2453	0.03284	0.144	0.78641	-0.3467	0.19247	0.48	0.8227	-0.2816	0.27608	0.81	0.8653	-0.2087	0.13874	0.46	0.83859	-0.254	0.01416	0.09	0.81871	-0.2886	0.02866	0.1	0.82422	-0.2789	0.04657	0.18	0.83399	-0.2619	0.11286	0.49	0.85363	-0.2283
Lipidomics	PE(18:0/18:3)	0.00898	0.04	0.71237	-0.4893	0.03405	0.144	0.63972	-0.6445	0.23609	0.51	0.70878	-0.4966	0.24634	0.76	0.75183	-0.4115	0.06388	0.41	0.6793	-0.5579	0.01608	0.1	0.69168	-0.5318	0.01699	0.08	0.649	-0.6237	0.0349	0.16	0.70288	-0.5087	0.08765	0.45	0.7246	-0.4647
Lipidomics	PC(18:0/22:6)	0.0004	0.01	0.81161	-0.3011	0.03478	0.144	0.73809	-0.4381	0.82633	0.91	0.96678	-0.0487	0.04182	0.5	0.78032	-0.3579	0.07774	0.41	0.85216	-0.2308	0.00221	0.04	0.75438	-0.4066	0.10291	0.23	0.89114	-0.1663	0.02635	0.14	0.80854	-0.3066	0.00527	0.16	0.81466	-0.2957
Lipidomics	PC(16:0/14:0)	0.0356	0.12	0.82009	-0.2862	0.03545	0.144	0.76566	-0.3852	0.23284	0.51	0.78428	-0.3506	0.36542	0.86	0.80181	-0.3187	0.12047	0.43	0.80663	-0.31	0.0326	0.13	0.7832	-0.3525	0.03113	0.1	0.78975	-0.3405	0.14727	0.36	0.83553	-0.2592	0.13548	0.49	0.80378	-0.3151
Lipidomics	PC(18:2/22:6)	0.7837	0.88	0.91292	-0.1314	0.03575	0.144	0.74706	-0.4207	0.79101	0.91	0.98289	-0.0249	0.96865	0.98	0.95307	-0.0693	0.1552	0.46	0.80234	-0.3177	0.15661	0.29	0.83553	-0.2592	0.59977	0.73	0.88686	-0.1732	0.76757	0.89	0.93156	-0.1023	0.43278	0.68	0.89358	-0.1623
Lipidomics	PC(14:0/20:4)	0.01008	0.05	0.8108	-0.3026	0.03602	0.144	0.73061	-0.4528	0.25225	0.52	0.83304	-0.2635	0.16018	0.73	0.81762	-0.2905	0.14521	0.46	0.80106	-0.32	0.0086	0.07	0.76868	-0.3795	0.04095	0.12	0.80795	-0.3077	0.07392	0.25	0.81103	-0.3022	0.07251	0.44	0.81056	-0.303
Lipidomics	PE(18:0/18:2)	0.04782	0.14	0.82383	-0.2796	0.03622	0.144	0.71434	-0.4853	0.19948	0.48	0.81699	-0.2916	0.64925	0.91	0.89322	-0.1629	0.11985	0.43	0.78613	-0.3472	0.05825	0.18	0.7927	-0.3352	0.02285	0.09	0.77666	-0.3647	0.09038	0.27	0.8028	-0.3169	0.23924	0.55	0.84893	-0.2363
Lipidomics	TAG52:4-FA18:2	0.00131	0.02	0.53601	-0.8997	0.03629	0.144	0.59392	-0.7517	0.01214	0.16	0.18614	-2.4255	0.71995	0.91	0.82391	-0.2794	0.03173	0.4	0.34741	-1.5253	0.05633	0.18	0.68764	-0.5403	0.0006	0.01	0.27573	-1.8587	0.00799	0.08	0.48783	-1.0356	0.07121	0.44	0.59314	-0.7536
Lipidomics	PC(18:0/22:5)	0.0631	0.17	0.84606	-0.2412	0.03653	0.144	0.74909	-0.4168	0.74109	0.91	0.91388	-0.1299	0.16106	0.73	0.8087	-0.3063	0.35861	0.61	0.87138	-0.1986	0.00934	0.07	0.77587	-0.3661	0.41629	0.59	0.90029	-0.1515	0.30135	0.57	0.85611	-0.2241	0.11644	0.49	0.83619	-0.2581
Lipidomics	TAG52:3-FA16:0	0.00238	0.02	0.51824	-0.9483	0.03665	0.144	0.56176	-0.832	0.01277	0.16	0.18421	-2.4406	0.67305	0.91	0.74999	-0.4151	0.04537	0.4	0.32918	-1.603	0.06418	0.18	0.63957	-0.6448	0.00089	0.01	0.26507	-1.9156	0.01084	0.09	0.48574	-1.0418	0.08957	0.45	0.55662	-0.8452
Lipidomics	PC(16:0/18:0)	0.00251	0.02	0.81626	-0.2929	0.03669	0.144	0.76964	-0.3778	0.24988	0.52	0.84669	-0.2401	0.10391	0.61	0.79729	-0.3268	0.11796	0.43	0.82296	-0.2811	0.00551	0.05	0.78099	-0.3566	0.02987	0.1	0.82359	-0.28	0.03555	0.16	0.82373	-0.2798	0.03573	0.4	0.80858	-0.3065
Lipidomics	PE(16:0/20:4)	0.04033	0.13	0.76267	-0.3909	0.03715	0.144	0.66819	-0.5817	0.26917	0.54	0.74481	-0.4251	0.52047	0.89	0.78952	-0.341	0.13285	0.45	0.74198	-0.4306	0.05378	0.17	0.72468	-0.4646	0.03557	0.11	0.7274	-0.4592	0.11718	0.33	0.75623	-0.4031	0.17759	0.53	0.77025	-0.3766
Lipidomics	TAG52:5-FA16:0	0.00023	0.01	0.44699	-1.1617	0.03719	0.144	0.52391	-0.9326	0.00372	0.13	0.1542	-2.6971	0.32029	0.84	0.59141	-0.7578	0.02479	0.38	0.26804	-1.8995	0.02202	0.11	0.55461	-0.8504	0.00016	0.01	0.21928	-2.1892	0.00373	0.07	0.4496	-1.1533	0.02462	0.4	0.44402	-1.1713
Lipidomics	PC(18:0/18:0)	9.2E-05	0	0.80633	-0.3106	0.03786	0.145	0.77099	-0.3752	0.62593	0.83	0.93223	-0.1012	0.02708	0.5	0.78321	-0.3525	0.04303	0.4	0.83122	-0.2667	0.0017	0.04	0.77067	-0.3758	0.03888	0.12	0.86173	-0.2147	0.01473	0.11	0.80625	-0.3107	0.00184	0.16	0.80642	-0.3104
Lipidomics	PC(18:0/18:1)	0.00566	0.04	0.82363	-0.2799	0.0403	0.15	0.78197	-0.3548	0.19327	0.48	0.83087	-0.2673	0.17361	0.73	0.8401	-0.2514	0.10292	0.42	0.77479	-0.3681	0.00967	0.07	0.8054	-0.3122	0.02287	0.09	0.79377	-0.3332	0.05242	0.19	0.83626	-0.258	0.0566	0.41	0.81099	-0.3022
Lipidomics	TAG50:3-FA16:0	0.00848	0.04	0.55055	-0.8611	0.04033	0.15	0.54746	-0.8692	0.01331	0.16	0.29924	-1.7406	0.37373	0.86	0.61697	-0.6967	0.09014	0.42	0.35284	-1.5029	0.02632	0.12	0.57919	-0.7879	0.00404	0.04	0.35068	-1.5118	0.04912	0.18	0.59471	-0.7497	0.08751	0.45	0.50757	-0.9783
Lipidomics	PC(18:0/20:5)	9E-06	0	0.65429	-0.612	0.0418	0.151	0.67138	-0.5748	0.0686	0.28	0.62182	-0.6854	0.02316	0.5	0.68567	-0.5444	0.01113	0.32	0.62717	-0.6731	0.00215	0.04	0.68036	-0.5556	0.00093	0.01	0.59626	-0.746	0.00282	0.07	0.65028	-0.6209	0.00089	0.11	0.65901	-0.6016
Lipidomics	PE(O-18:0/20:3)	0.05288	0.15	0.83188	-0.2656	0.04217	0.151	0.64387	-0.6352	0.96967	0.99	0.975	-0.0365	0.82048	0.93	1.0114	0.0163	0.07327	0.41	0.75809	-0.3996	0.07735	0.2	0.78875	-0.3424	0.12071	0.25	0.82553	-0.2766	0.08916	0.27	0.77457	-0.3685	0.36672	0.66	0.899	-0.1536
Lipidomics	LPE(18:3)	9.8E-06	0	0.57818	-0.7904	0.04261	0.151	0.63	-0.6666	0.00084	0.11	0.3725	-1.4247	0.17479	0.73	0.78038	-0.3578	0.00303	0.19	0.4192	-1.2543	0.01322	0.09	0.6968	-0.5212	9.8E-06	0	0.37112	-1.4301	0.00032	0.03	0.55231	-0.8565	0.00405	0.16	0.6135	-0.7049
Lipidomics	PC(18:0/22:4)	0.06401	0.17	0.87071	-0.1997	0.04374	0.153	0.79072	-0.3388	0.58067	0.8	0.90326	-0.1468	0.59137	0.89	0.88249	-0.1803	0.2396	0.51	0.86294	-0.2127	0.07383	0.19	0.83413	-0.2617	0.20818	0.39	0.8857	-0.1751	0.17726	0.41	0.86831	-0.2037	0.22178	0.55	0.87317	-0.1957
Lipidomics	LPC(16:0)	0.75303	0.86	0.97404	-0.038	0.04447	0.154	0.84516	-0.2427	0.2548	0.52	1.1546	0.20734	0.74579	0.91	0.94726	-0.0782	0.88951	0.93	1.0075	0.01082	0.1095	0.23	0.88508	-0.1761	0.50269	0.65	1.0656	0.0917	0.92031	0.99	0.97451	-0.0373	0.75058	0.88	0.97358	-0.0386
Lipidomics	PE(P-16:0/16:0)	0.00703	0.04	0.73534	-0.4435	0.04757	0.157	0.55119	-0.8594	0.42498	0.67	0.80787	-0.3078	0.38713	0.86	0.8431	-0.2462	0.1659	0.47	0.80861	-0.3065	0.02333	0.11	0.66797	-0.5821	0.1455	0.28	0.8134	-0.298	0.02338	0.13	0.64114	-0.6413	0.12683	0.49	0.82769	-0.2728
Lipidomics	PC(18:1/22:6)	0.35721	0.54	0.88679	-0.1733	0.04801	0.157	0.76312	-0.39	0.25842	0.53	0.83048	-0.268	0.58285	0.89	0.87365	-0.1949	0.55758	0.74	0.90893	-0.1378	0.0726	0.19	0.80981	-0.3044	0.33073	0.52	0.89827	-0.1548	0.55091	0.8	0.88628	-0.1742	0.49848	0.71	0.8873	-0.1725
Lipidomics	PA 18_1-18_2_2	0.0073	0.04	0.85884	-0.2195	0.04807	0.157	0.82811	-0.2721	0.02369	0.19	0.74717	-0.4205	0.26393	0.79	0.86764	-0.2048	0.31003	0.58	0.88439	-0.1773	0.02303	0.11	0.8471	-0.2394	0.02408	0.09	0.8308	-0.2674	0.02333	0.13	0.84307	-0.2463	0.14038	0.49	0.87532	-0.1921
Lipidomics	PE(O-16:0/20:4)	0.00915	0.04	0.81805	-0.2897	0.04812	0.157	0.76025	-0.3955	0.28275	0.54	0.84486	-0.2432	0.65318	0.91	0.88669	-0.1735	0.0589	0.41	0.79185	-0.3367	0.08145	0.2	0.81392	-0.2971	0.01661	0.08	0.78672	-0.3461	0.01798	0.12	0.795	-0.331	0.15443	0.52	0.84401	-0.2447
Lipidomics	PE(O-18:0/22:6)	0.00381	0.03	0.79407	-0.3327	0.04836	0.157	0.71769	-0.4786	0.18035	0.48	0.80866	-0.3064	0.42242	0.86	0.91251	-0.1321	0.0661	0.41	0.75734	-0.401	0.03241	0.13	0.80016	-0.3216	0.01468	0.07	0.7399	-0.4346	0.01103	0.09	0.75362	-0.4081	0.09906	0.49	0.84402	-0.2447
Lipidomics	PA 16_1-18_1_2	0.0136	0.06	0.88157	-0.1819	0.05116	0.158	0.85854	-0.2201	0.03026	0.2	0.78907	-0.3418	0.42686	0.86	0.89959	-0.1527	0.22558	0.51	0.87327	-0.1955	0.05009	0.17	0.87691	-0.1895	0.02523	0.09	0.84832	-0.2373	0.03969	0.17	0.876	-0.191	0.15739	0.52	0.88716	-0.1727
Lipidomics	PC(17:0/18:1)	0.06863	0.17	0.85436	-0.2271	0.05117	0.158	0.80503	-0.3129	0.32465	0.58	0.82652	-0.2749	0.19346	0.73	0.84793	-0.238	0.38685	0.65	0.84399	-0.2447	0.01399	0.09	0.82193	-0.2829	0.13451	0.27	0.82513	-0.2773	0.17325	0.41	0.86213	-0.214	0.22972	0.55	0.84631	-0.2408
Lipidomics	PE(O-16:0/22:6)	0.01202	0.05	0.8218	-0.2831	0.05867	0.171	0.78128	-0.3561	0.19128	0.48	0.81354	-0.2977	0.5552	0.89	0.9065	-0.1416	0.04978	0.41	0.72418	-0.4656	0.0651	0.18	0.83279	-0.264	0.00903	0.06	0.73743	-0.4394	0.0347	0.16	0.81642	-0.2926	0.11941	0.49	0.82765	-0.2729
Lipidomics	PC(16:0/16:1)	0.23053	0.41	0.73285	-0.4484	0.0594	0.171	0.70216	-0.5101	0.06417	0.28	0.67968	-0.5571	0.32253	0.84	0.61953	-0.6908	0.2204	0.51	0.54785	-0.8682	0.04499	0.16	0.67155	-0.5744	0.05038	0.14	0.61336	-0.7052	0.67271	0.85	0.87811	-0.1875	0.22234	0.55	0.59702	-0.7442
Lipidomics	PC(18:1/20:5)	0.01472	0.06	0.74739	-0.4201	0.06275	0.177	0.75739	-0.4009	0.05055	0.26	0.5385	-0.893	0.39102	0.86	0.80971	-0.3045	0.04568	0.4	0.59554	-0.7477	0.05029	0.17	0.78224	-0.3543	0.00237	0.03	0.5587	-0.8399	0.08395	0.26	0.76827	-0.3803	0.08328	0.45	0.72424	-0.4655
Lipidomics	PE(18:1/16:1)	0.04308	0.13	0.82031	-0.2858	0.06349	0.177	0.72797	-0.4581	0.06019	0.28	0.70464	-0.5051	0.54728	0.89	0.88575	-0.175	0.18143	0.47	0.8111	-0.302	0.07994	0.2	0.80009	-0.3218	0.0148	0.07	0.75692	-0.4018	0.10003	0.29	0.79235	-0.3358	0.2233	0.55	0.85375	-0.2281
Lipidomics	PE(18:0/20:5)	0.01276	0.05	0.74531	-0.4241	0.0661	0.182	0.69587	-0.5231	0.32418	0.58	0.7737	-0.3702	0.16808	0.73	0.73355	-0.447	0.05281	0.41	0.64583	-0.6308	0.02271	0.11	0.72078	-0.4724	0.01598	0.08	0.68319	-0.5496	0.1604	0.39	0.7879	-0.3439	0.03411	0.4	0.69732	-0.5201
Lipidomics	PE(O-16:0/22:5)	0.05629	0.16	0.81418	-0.2966	0.06713	0.182	0.72259	-0.4687	0.44911	0.68	0.87858	-0.1868	0.52516	0.89	0.85592	-0.2244	0.07348	0.41	0.70008	-0.5144	0.06651	0.18	0.7821	-0.3546	0.02856	0.1	0.7566	-0.4024	0.24425	0.5	0.83449	-0.261	0.13038	0.49	0.79184	-0.3367
Lipidomics	HCER(22:0)	0.00051	0.01	0.68436	-0.5472	0.06752	0.182	0.62608	-0.6756	0.00604	0.13	0.53765	-0.8953	0.03389	0.5	0.62702	-0.6734	0.32127	0.59	0.88065	-0.1834	0.00479	0.05	0.62962	-0.6674	0.01062	0.06	0.71561	-0.4828	0.00732	0.08	0.64916	-0.6234	0.03375	0.4	0.72454	-0.4649
Lipidomics	TAG54:2-FA20:0	0.00098	0.01	0.48862	-1.0332	0.07282	0.194	0.59865	-0.7402	0.0015	0.11	0.16987	-2.5575	0.38209	0.86	0.59332	-0.7531	0.0654	0.41	0.30207	-1.7271	0.04451	0.16	0.59058	-0.7598	0.00081	0.01	0.27857	-1.8439	0.00958	0.09	0.53542	-0.9013	0.04971	0.41	0.44977	-1.1527
Lipidomics	PC(15:0/20:3)	0.02584	0.1	0.86105	-0.2158	0.0774	0.203	0.79444	-0.332	0.28126	0.54	0.86918	-0.2023	0.19175	0.73	0.86023	-0.2172	0.35086	0.61	0.89284	-0.1635	0.02171	0.11	0.81818	-0.2895	0.13263	0.27	0.87103	-0.1992	0.06614	0.23	0.84837	-0.2372	0.19816	0.53	0.87397	-0.1943
Lipidomics	PE(18:0/20:4)	0.05359	0.15	0.84671	-0.2401	0.07755	0.203	0.74832	-0.4183	0.2259	0.5	0.83807	-0.2549	0.75628	0.91	0.91335	-0.1308	0.08406	0.41	0.79686	-0.3276	0.11103	0.23	0.82169	-0.2833	0.02129	0.09	0.81025	-0.3036	0.16515	0.4	0.83412	-0.2617	0.18882	0.53	0.86064	-0.2165
Lipidomics	PC(16:0/20:1)	0.44712	0.64	0.84379	-0.245	0.08323	0.215	0.792	-0.3364	0.06048	0.28	0.68955	-0.5363	0.47577	0.86	0.77772	-0.3627	0.18557	0.47	0.63702	-0.6506	0.10352	0.23	0.78806	-0.3436	0.03822	0.12	0.67439	-0.5683	0.98976	0.99	0.95965	-0.0594	0.30814	0.61	0.73302	-0.4481
Lipidomics	PC(18:0/20:3)	0.08729	0.21	0.86854	-0.2033	0.08725	0.221	0.77172	-0.3739	0.79316	0.91	1.0195	0.0278	0.08955	0.61	0.78207	-0.3546	0.5612	0.74	0.92068	-0.1192	0.01261	0.09	0.77763	-0.3628	0.79601	0.89	0.96903	-0.0454	0.42416	0.71	0.89515	-0.1598	0.10989	0.49	0.84282	-0.2467
Lipidomics	TAG51:4-FA18:2	0.0065	0.04	0.61489	-0.7016	0.08729	0.221	0.56655	-0.8197	0.00536	0.13	0.2447	-2.0309	0.6081	0.89	0.86504	-0.2092	0.17334	0.47	0.56485	-0.8241	0.08338	0.2	0.68492	-0.546	0.0039	0.04	0.43158	-1.2123	0.01865	0.12	0.53288	-0.9081	0.17127	0.52	0.72219	-0.4696
Lipidomics	PC(18:0/20:4)	0.00165	0.02	0.84328	-0.2459	0.09459	0.235	0.80472	-0.3134	0.18897	0.48	0.84904	-0.2361	0.24532	0.76	0.88304	-0.1795	0.14906	0.46	0.90508	-0.1439	0.0325	0.13	0.83857	-0.254	0.0357	0.11	0.87781	-0.188	0.01023	0.09	0.79431	-0.3322	0.08093	0.45	0.89458	-0.1607
Lipidomics	PC(18:1/18:3)	0.06496	0.17	1.0896	0.12374	0.09541	0.235	1.2526	0.32498	0.76997	0.91	0.84149	-0.249	0.14048	0.71	1.2723	0.34748	0.55635	0.74	0.72969	-0.4546	0.03254	0.13	1.2416	0.31215	0.93367	0.96	0.7718	-0.3737	0.08893	0.27	1.1651	0.22042	0.4352	0.68	1.0215	0.03072
Lipidomics	PG 18_1-18_2_2	0.22746	0.41	0.9173	-0.1245	0.09672	0.235	0.84183	-0.2484	0.0834	0.31	0.78905	-0.3418	0.22971	0.75	0.891	-0.1665	0.8234	0.92	1.0418	0.05908	0.0351	0.13	0.86543	-0.2085	0.37591	0.56	0.92473	-0.1129	0.18115	0.41	0.88485	-0.1765	0.68982	0.85	0.95298	-0.0695
Lipidomics	PC(18:2/20:2)	0.42269	0.62	0.89064	-0.1671	0.09713	0.235	0.81001	-0.304	0.08205	0.31	0.6729	-0.5715	0.94824	0.98	0.94269	-0.0851	0.30934	0.58	0.80661	-0.3101	0.37857	0.51	0.86798	-0.2043	0.04166	0.12	0.74456	-0.4255	0.51059	0.77	0.88865	-0.1703	0.6235	0.8	0.89283	-0.1635
Lipidomics	HCER(20:0)	0.05829	0.16	0.7869	-0.3457	0.10146	0.238	0.65987	-0.5998	0.788	0.91	0.81361	-0.2976	0.53413	0.89	0.8217	-0.2833	0.93865	0.96	1.0925	0.12758	0.0671	0.18	0.72182	-0.4703	0.753	0.86	0.92327	-0.1152	0.0392	0.17	0.64124	-0.6411	0.69478	0.86	0.95553	-0.0656
Lipidomics	PC(18:1/20:1)	0.32191	0.51	0.85197	-0.2311	0.10514	0.242	0.80953	-0.3049	0.17667	0.48	0.56964	-0.8119	0.80105	0.92	0.90114	-0.1502	0.04064	0.4	0.44557	-1.1663	0.20382	0.35	0.84808	-0.2377	0.01008	0.06	0.49925	-1.0022	0.9845	0.99	0.95838	-0.0613	0.2031	0.53	0.75062	-0.4139
Lipidomics	PG 18_0-20_4_2	0.0757	0.19	0.8502	-0.2341	0.1072	0.242	0.79319	-0.3343	0.04119	0.23	0.73465	-0.4449	0.22649	0.75	0.87666	-0.1899	0.87581	0.93	0.94335	-0.0841	0.03343	0.13	0.82743	-0.2733	0.23187	0.41	0.82419	-0.279	0.02406	0.13	0.80255	-0.3173	0.56628	0.76	0.90402	-0.1456
Lipidomics	FFA(18:4)	0.00096	0.01	0.69873	-0.5172	0.1083	0.242	0.63485	-0.6555	0.02182	0.19	0.64208	-0.6392	0.00359	0.46	0.71528	-0.4834	0.58524	0.77	0.87364	-0.1949	0.00752	0.06	0.67457	-0.568	0.04711	0.13	0.71388	-0.4863	0.00638	0.08	0.63617	-0.6525	0.05833	0.41	0.78293	-0.353
Lipidomics	DAG(16:0/22:6)	0.00592	0.04	0.70584	-0.5026	0.1086	0.242	0.69375	-0.5275	0.01391	0.16	0.47284	-1.0806	0.20903	0.73	0.7801	-0.3583	0.88265	0.93	0.95555	-0.0656	0.03332	0.13	0.73253	-0.449	0.08799	0.2	0.66814	-0.5818	0.00237	0.07	0.5947	-0.7498	0.49911	0.71	0.85734	-0.2221
Lipidomics	PE(P-18:0/18:2)	0.03474	0.12	0.80262	-0.3172	0.11051	0.244	0.74703	-0.4208	0.36369	0.62	0.82215	-0.2825	0.61633	0.89	0.84992	-0.2346	0.07655	0.41	0.73608	-0.4421	0.10784	0.23	0.79284	-0.3349	0.02682	0.09	0.74196	-0.4306	0.09904	0.29	0.80279	-0.3169	0.171	0.52	0.80243	-0.3176
Lipidomics	FFA(20:4)	0.0105	0.05	0.74884	-0.4173	0.116	0.252	0.65311	-0.6146	0.10669	0.34	0.68566	-0.5444	0.07546	0.59	0.72388	-0.4662	0.83035	0.92	1.0216	0.03087	0.01687	0.1	0.68534	-0.5451	0.3295	0.52	0.84075	-0.2503	0.01403	0.11	0.65939	-0.6008	0.31739	0.62	0.85655	-0.2234
Lipidomics	LPE(18:1)	0.03811	0.12	0.78879	-0.3423	0.1263	0.264	0.76122	-0.3936	0.05751	0.28	0.63003	-0.6665	0.24318	0.76	0.80326	-0.3161	0.32678	0.59	0.7619	-0.3923	0.06237	0.18	0.78831	-0.3432	0.03768	0.11	0.69531	-0.5243	0.11532	0.32	0.79141	-0.3375	0.16528	0.52	0.78573	-0.3479
Lipidomics	LPE(20:1)	0.57043	0.73	0.89497	-0.1601	0.12661	0.264	0.82309	-0.2809	0.01586	0.16	0.66899	-0.5799	0.47618	0.86	0.82939	-0.2699	0.39877	0.65	0.73114	-0.4518	0.1833	0.32	0.83726	-0.2563	0.06056	0.16	0.71674	-0.4805	0.97598	0.99	0.98383	-0.0235	0.43031	0.68	0.79807	-0.3254
Lipidomics	TAG54:5-FA18:1	0.04233	0.13	0.60578	-0.7231	0.13787	0.282	0.6617	-0.5958	0.01422	0.16	0.27596	-1.8575	0.4591	0.86	0.88887	-0.17	0.12285	0.43	0.4795	-1.0604	0.79552	0.88	0.75532	-0.4048	0.00465	0.04	0.37312	-1.4223	0.01058	0.09	0.53763	-0.8953	0.45525	0.69	0.69231	-0.5305
Lipidomics	PC(18:2/20:4)	0.27037	0.46	0.8871	-0.1728	0.14661	0.296	0.81484	-0.2954	0.13082	0.38	0.74539	-0.4239	0.90027	0.96	0.95642	-0.0643	0.15992	0.46	0.78162	-0.3555	0.24156	0.39	0.87886	-0.1863	0.0214	0.09	0.74841	-0.4181	0.45112	0.73	0.88633	-0.1741	0.41541	0.68	0.88798	-0.1714
Lipidomics	FFA(18:1)	0.00566	0.04	0.7357	-0.4428	0.15549	0.309	0.73442	-0.4453	0.00882	0.16	0.51713	-0.9514	0.02255	0.5	0.70919	-0.4958	0.95345	0.97	0.9662	-0.0496	0.01462	0.09	0.7264	-0.4612	0.07711	0.18	0.7083	-0.4976	0.00761	0.08	0.67543	-0.5661	0.25509	0.56	0.81119	-0.3019
Lipidomics	PC(18:1/18:2)	0.16256	0.32	0.89301	-0.1633	0.16431	0.321	0.86593	-0.2077	0.02316	0.19	0.66384	-0.5911	0.88226	0.95	0.99119	-0.0128	0.15063	0.46	0.80313	-0.3163	0.36201	0.5	0.91939	-0.1213	0.00527	0.04	0.73042	-0.4532	0.19471	0.44	0.87424	-0.1939	0.46283	0.69	0.9138	-0.1301
Lipidomics	LPE(18:2)	0.00214	0.02	0.69499	-0.5249	0.17786	0.342	0.72074	-0.4724	0.00509	0.13	0.4306	-1.2156	0.46226	0.86	0.88565	-0.1752	0.01487	0.36	0.50999	-0.9715	0.13728	0.27	0.796	-0.3292	1E-04	0.01	0.45758	-1.1279	0.02194	0.13	0.6801	-0.5562	0.03493	0.4	0.71414	-0.4857
Lipidomics	FFA(16:1)	0.10335	0.24	0.80581	-0.3115	0.18554	0.354	0.7315	-0.4511	0.04895	0.26	0.59833	-0.741	0.04337	0.5	0.67108	-0.5754	0.71374	0.85	1.1708	0.22749	0.02341	0.11	0.71239	-0.4893	0.39889	0.59	0.88331	-0.179	0.1139	0.32	0.77728	-0.3635	0.47523	0.7	0.83933	-0.2527
Lipidomics	TAG50:1-FA16:0	0.01168	0.05	0.57724	-0.7928	0.21763	0.401	0.66238	-0.5943	0.00219	0.11	0.19468	-2.3608	0.36017	0.86	0.56804	-0.8159	0.23315	0.51	0.51689	-0.9521	0.18162	0.32	0.63544	-0.6542	0.00745	0.05	0.41401	-1.2723	0.04014	0.17	0.60864	-0.7163	0.12437	0.49	0.54406	-0.8782
Lipidomics	FFA(18:2)	0.00389	0.03	0.74889	-0.4172	0.23332	0.425	0.7841	-0.3509	0.00883	0.16	0.54614	-0.8727	0.01944	0.5	0.76024	-0.3955	0.99462	1	0.94928	-0.0751	0.02852	0.12	0.77542	-0.367	0.06039	0.16	0.70735	-0.4995	0.00647	0.08	0.67561	-0.5657	0.23005	0.55	0.84262	-0.247
Lipidomics	DAG(18:0/22:6)	0.04095	0.13	0.81414	-0.2967	0.2614	0.47	0.75491	-0.4056	0.12641	0.38	0.72521	-0.4635	0.0837	0.61	0.7905	-0.3392	0.63814	0.81	1.1115	0.15255	0.0617	0.18	0.77175	-0.3738	0.43121	0.6	0.87862	-0.1867	0.04073	0.17	0.72052	-0.4729	0.52835	0.74	0.93146	-0.1024
Lipidomics	PE(O-18:0/16:0)	0.03479	0.12	0.87306	-0.1959	0.26273	0.47	0.86329	-0.2121	0.18548	0.48	0.82084	-0.2848	0.67722	0.91	0.95257	-0.0701	0.08148	0.41	0.78155	-0.3556	0.23942	0.39	0.90543	-0.1433	0.01423	0.07	0.78194	-0.3549	0.14761	0.36	0.87436	-0.1937	0.12335	0.49	0.87163	-0.1982
Lipidomics	FFA(22:2)	0.00903	0.04	0.76474	-0.387	0.26586	0.473	0.79601	-0.3291	0.07117	0.28	0.65873	-0.6023	0.02358	0.5	0.76468	-0.3871	0.64005	0.81	0.93132	-0.1027	0.03653	0.14	0.78268	-0.3535	0.10123	0.23	0.7357	-0.4428	0.02311	0.13	0.71014	-0.4938	0.16343	0.52	0.83543	-0.2594
Lipidomics	PE(P-16:0/22:4)	0.06138	0.17	1.1433	0.19316	0.27297	0.479	1.1229	0.16718	0.29021	0.55	1.2349	0.30441	0.09673	0.61	1.203	0.2666	0.92742	0.96	0.9886	-0.0165	0.04955	0.17	1.1645	0.21974	0.77243	0.86	1.0517	0.07273	0.07942	0.26	1.1778	0.23614	0.35604	0.65	1.1041	0.14284
Lipidomics	TAG53:2-FA18:2	0.00314	0.03	0.52667	-0.925	0.2934	0.488	0.51307	-0.9628	0.00093	0.11	0.22099	-2.178	0.61899	0.89	1.1623	0.21698	0.03648	0.4	0.31504	-1.6664	0.55858	0.69	0.72843	-0.4571	0.00017	0.01	0.28455	-1.8133	0.00828	0.08	0.44855	-1.1567	0.13832	0.49	0.62677	-0.674
Lipidomics	FFA(14:0)	0.11857	0.26	0.89377	-0.162	0.30321	0.491	0.83064	-0.2677	0.03446	0.22	0.65233	-0.6163	0.03304	0.5	0.77587	-0.3661	0.39198	0.65	1.4438	0.52988	0.05128	0.17	0.80949	-0.3049	0.75885	0.86	1.026	0.03706	0.03058	0.15	0.75554	-0.4044	0.93556	0.97	1.0637	0.08904
Lipidomics	PG 16_0-20_3	0.02043	0.08	0.88679	-0.1733	0.35859	0.549	0.91522	-0.1278	0.01498	0.16	0.65858	-0.6026	0.04493	0.5	0.86836	-0.2036	0.76963	0.88	1.0016	0.00225	0.06331	0.18	0.88632	-0.1741	0.06587	0.16	0.85475	-0.2264	0.04333	0.17	0.84687	-0.2398	0.25283	0.56	0.92734	-0.1088
Lipidomics	FFA(16:0)	0.03583	0.12	0.82329	-0.2805	0.40981	0.614	0.84637	-0.2406	0.03046	0.2	0.6629	-0.5931	0.02418	0.5	0.7833	-0.3524	0.70977	0.85	1.0487	0.06854	0.07565	0.2	0.8182	-0.2895	0.25969	0.43	0.82792	-0.2724	0.03852	0.17	0.75908	-0.3977	0.45226	0.69	0.8999	-0.1522
Lipidomics	FFA(17:0)	0.15771	0.32	0.88594	-0.1747	0.45915	0.674	0.86937	-0.202	0.06007	0.28	0.6828	-0.5505	0.05783	0.55	0.82796	-0.2724	0.3758	0.63	1.2924	0.3701	0.12501	0.25	0.85122	-0.2324	0.72998	0.85	0.96024	-0.0585	0.04685	0.18	0.75926	-0.3973	0.93353	0.97	1.0412	0.05824
Lipidomics	LPC(20:5)	0.00107	0.01	0.78863	-0.3426	0.4609	0.674	0.92541	-0.1118	0.19145	0.48	0.73406	-0.446	0.32888	0.85	0.89788	-0.1554	0.00139	0.19	0.63607	-0.6528	0.17678	0.31	0.89788	-0.1554	0.00083	0.01	0.65391	-0.6129	0.06744	0.23	0.80657	-0.3101	0.00565	0.16	0.77055	-0.376
Lipidomics	LPE(20:3)	0.43908	0.64	0.929	-0.1063	0.5429	0.768	0.86767	-0.2048	0.1688	0.48	0.78524	-0.3488	0.92525	0.97	1.055	0.07723	0.06888	0.41	0.63697	-0.6507	0.78038	0.88	0.95161	-0.0716	0.01938	0.09	0.70135	-0.5118	0.9117	0.99	0.98358	-0.0239	0.2656	0.58	0.87224	-0.1972
Lipidomics	HCER(22:1)	0.48927	0.67	0.86832	-0.2037	0.54695	0.769	0.81459	-0.2959	0.0957	0.33	0.65809	-0.6037	0.52012	0.89	0.78982	-0.3404	0.27057	0.54	0.64509	-0.6324	0.33748	0.48	0.80099	-0.3201	0.04775	0.13	0.65229	-0.6164	0.96884	0.99	0.98875	-0.0163	0.38765	0.68	0.75246	-0.4103
Lipidomics	CE(20:5)	0.036	0.12	0.86012	-0.2174	0.55627	0.778	1.0704	0.09817	0.38803	0.65	0.86641	-0.2069	0.07155	0.58	0.73928	-0.4358	0.18555	0.47	0.82354	-0.2801	0.2207	0.36	0.87469	-0.1932	0.07061	0.17	0.82879	-0.2709	0.66155	0.85	0.95713	-0.0632	0.02645	0.4	0.77668	-0.3646
Lipidomics	PI 14_0-18_0_2	0.02946	0.11	0.86457	-0.2099	0.57368	0.786	0.96656	-0.0491	0.40719	0.66	0.93523	-0.0966	0.94938	0.98	1.0043	0.00623	0.00178	0.19	0.53864	-0.8926	0.98782	0.99	0.98897	-0.016	0.001	0.02	0.67044	-0.5768	0.35537	0.63	0.95993	-0.059	0.03406	0.4	0.76444	-0.3875
Lipidomics	PE(P-18:0/22:5)	0.54093	0.71	0.9452	-0.0813	0.58103	0.786	0.87881	-0.1864	0.14322	0.41	0.78147	-0.3557	0.20368	0.73	1.1475	0.19847	0.14262	0.46	0.79881	-0.3241	0.80925	0.88	0.99347	-0.0095	0.02104	0.09	0.76455	-0.3873	0.48565	0.76	0.90674	-0.1412	0.86126	0.95	0.99269	-0.0106
Lipidomics	PE(P-18:0/20:4)	0.23571	0.42	0.9117	-0.1334	0.5864	0.786	0.89756	-0.1559	0.6047	0.83	0.91307	-0.1312	0.60386	0.89	1.068	0.09496	0.06906	0.41	0.81809	-0.2897	0.89366	0.95	0.9708	-0.0428	0.04644	0.13	0.82036	-0.2857	0.30083	0.57	0.88173	-0.1816	0.53418	0.74	0.94717	-0.0783
Lipidomics	SM d18_1-18_1	0.33765	0.52	1.1174	0.16019	0.59586	0.786	1.0655	0.09154	0.11536	0.36	1.3829	0.46769	0.52767	0.89	0.86356	-0.2116	0.04056	0.4	1.3803	0.46494	0.93577	0.97	0.97353	-0.0387	0.0059	0.04	1.3886	0.47361	0.50186	0.77	1.0792	0.10997	0.50524	0.71	1.1538	0.20633
Lipidomics	PE(P-18:0/20:5)	0.1658	0.32	0.88899	-0.1698	0.61075	0.802	0.9204	-0.1197	0.82308	0.91	0.91458	-0.1288	0.77229	0.91	1.0248	0.03531	0.01948	0.36	0.66651	-0.5853	0.80726	0.88	0.96429	-0.0525	0.02642	0.09	0.73588	-0.4425	0.5021	0.77	0.91597	-0.1266	0.20587	0.53	0.85893	-0.2194
Lipidomics	LPE(16:1)	0.29627	0.49	0.89844	-0.1545	0.64258	0.839	0.91778	-0.1238	0.20105	0.48	0.72555	-0.4629	0.29294	0.82	0.85734	-0.2221	0.105	0.42	0.69366	-0.5277	0.28559	0.44	0.89596	-0.1585	0.03163	0.1	0.7149	-0.4842	0.83259	0.94	1.0063	0.00907	0.11511	0.49	0.79297	-0.3347
Lipidomics	FFA(24:1)	0.28827	0.48	0.87266	-0.1965	0.64648	0.84	0.91463	-0.1287	0.09899	0.33	0.73456	-0.4451	0.04485	0.5	0.7947	-0.3315	0.29841	0.58	1.1764	0.23439	0.14271	0.28	0.85429	-0.2272	0.92368	0.96	0.91304	-0.1313	0.09168	0.27	0.79772	-0.326	0.89933	0.97	0.96123	-0.057
Lipidomics	HCER(16:0)	0.00741	0.04	0.63401	-0.6574	0.66511	0.842	0.89276	-0.1637	0.49128	0.72	0.79787	-0.3258	0.0437	0.5	0.55972	-0.8372	0.22561	0.51	0.60966	-0.7139	0.06189	0.18	0.72189	-0.4701	0.10484	0.23	0.66348	-0.5919	0.12293	0.34	0.67714	-0.5625	0.03013	0.4	0.59077	-0.7593
Lipidomics	SM d18_1-22_5	0.0477	0.14	0.88727	-0.1726	0.66776	0.842	1.0315	0.04479	0.452	0.68	0.89492	-0.1602	0.11702	0.62	0.79944	-0.3229	0.26075	0.54	0.88571	-0.1751	0.23191	0.38	0.89447	-0.1609	0.1458	0.28	0.88903	-0.1697	0.51691	0.77	0.94124	-0.0874	0.0451	0.41	0.84061	-0.2505
Lipidomics	SM d18_1-14_0	0.10707	0.24	0.85056	-0.2335	0.66801	0.842	1.1464	0.1971	0.99831	1	1.0165	0.02359	0.22408	0.75	0.70016	-0.5142	0.13186	0.45	0.72059	-0.4728	0.43316	0.57	0.88998	-0.1682	0.18198	0.35	0.83316	-0.2633	0.9601	0.99	1.0364	0.05164	0.04446	0.41	0.71072	-0.4927
Lipidomics	PE(P-18:0/22:4)	0.58638	0.74	1.0384	0.05442	0.68188	0.856	0.90123	-0.15	0.35484	0.61	0.86685	-0.2062	0.03953	0.5	1.3801	0.46479	0.69215	0.84	0.94679	-0.0789	0.31999	0.46	1.0909	0.12553	0.23934	0.42	0.88305	-0.1794	0.64367	0.84	0.93751	-0.0931	0.1901	0.53	1.172	0.22899
Lipidomics	DAG(18:1/22:5)	0.00903	0.04	0.65626	-0.6077	0.69141	0.862	0.78442	-0.3503	0.03705	0.22	0.45209	-1.1453	0.0018	0.46	0.49739	-1.0075	0.4853	0.71	0.77663	-0.3647	0.08179	0.2	0.64945	-0.6227	0.04569	0.13	0.64142	-0.6407	0.14565	0.36	0.69439	-0.5262	0.01156	0.27	0.61849	-0.6932
Lipidomics	PC(18:0/16:1)	0.94757	0.97	0.88285	-0.1798	0.69709	0.862	0.84759	-0.2386	0.03685	0.22	0.68321	-0.5496	0.28736	0.82	1.0708	0.09873	0.159	0.46	0.59471	-0.7497	0.29507	0.44	0.94555	-0.0808	0.02288	0.09	0.63451	-0.6563	0.84601	0.95	0.91456	-0.1289	0.92793	0.97	0.84875	-0.2366
Lipidomics	PG 18_0-20_4	0.0418	0.13	0.87241	-0.1969	0.70197	0.864	0.99538	-0.0067	0.01478	0.16	0.63628	-0.6523	0.04411	0.5	0.8516	-0.2318	0.33393	0.59	0.91755	-0.1242	0.55841	0.69	0.91433	-0.1292	0.0248	0.09	0.8227	-0.2816	0.21012	0.47	0.85974	-0.218	0.04274	0.41	0.88403	-0.1778
Lipidomics	LPE(20:4)	0.2097	0.39	0.90762	-0.1398	0.73096	0.894	0.90023	-0.1516	0.07182	0.28	0.70268	-0.5091	0.74426	0.91	1.1049	0.14395	0.03357	0.4	0.68306	-0.5499	0.95592	0.98	0.98704	-0.0188	0.00565	0.04	0.7055	-0.5033	0.60551	0.81	0.91845	-0.1227	0.22534	0.55	0.89693	-0.1569
Lipidomics	LPE(18:0)	0.08942	0.21	0.84164	-0.2487	0.74328	0.894	0.85762	-0.2216	0.19524	0.48	0.77123	-0.3748	0.99376	0.99	1.0326	0.04621	0.09932	0.42	0.7221	-0.4697	0.77009	0.88	0.93407	-0.0984	0.02266	0.09	0.73923	-0.4359	0.2703	0.54	0.82222	-0.2824	0.20584	0.53	0.86219	-0.2139
Lipidomics	LPE(20:2)	0.55693	0.72	0.95971	-0.0593	0.74841	0.894	0.90824	-0.1389	0.0899	0.32	0.66264	-0.5937	0.46935	0.86	1.1591	0.21303	0.08197	0.41	0.66888	-0.5802	0.6876	0.8	1.0208	0.02968	0.01419	0.07	0.6792	-0.5581	0.94443	0.99	0.98508	-0.0217	0.43131	0.68	0.9308	-0.1035
Lipidomics	SM d18_1-16_1	0.1592	0.32	0.87285	-0.1962	0.74916	0.894	0.92252	-0.1163	0.62033	0.83	1.087	0.12029	0.11089	0.61	0.71813	-0.4777	0.08426	0.41	0.80621	-0.3108	0.12975	0.26	0.80335	-0.3159	0.40392	0.59	0.91659	-0.1257	0.75208	0.88	1.025	0.03559	0.02841	0.4	0.7538	-0.4078
Lipidomics	PC(18:2/18:3)	0.56921	0.73	0.8813	-0.1823	0.75105	0.894	0.90228	-0.1484	0.44639	0.68	0.65421	-0.6122	0.68475	0.91	0.99821	-0.0026	0.01127	0.32	0.62374	-0.681	0.68631	0.8	0.94125	-0.0873	0.02267	0.09	0.61903	-0.6919	0.83138	0.94	0.91088	-0.1347	0.25281	0.56	0.84965	-0.2351
Lipidomics	LPE(16:0)	0.06261	0.17	0.84047	-0.2507	0.81633	0.908	0.88126	-0.1824	0.12509	0.38	0.73474	-0.4447	0.66601	0.91	0.9557	-0.0654	0.02382	0.38	0.64671	-0.6288	0.64274	0.76	0.91662	-0.1256	0.00324	0.04	0.6794	-0.5577	0.49493	0.77	0.87705	-0.1893	0.05561	0.41	0.80305	-0.3164
Lipidomics	CE(18:3)	0.03016	0.11	0.83924	-0.2528	0.89007	0.961	0.98017	-0.0289	0.22373	0.5	0.8039	-0.3149	0.126	0.66	0.71923	-0.4755	0.23845	0.51	0.83375	-0.2623	0.14688	0.28	0.84028	-0.2511	0.05507	0.15	0.80699	-0.3094	0.34151	0.61	0.91485	-0.1284	0.05182	0.41	0.76867	-0.3796
Lipidomics	PC(12:0/16:1)	0.30273	0.49	0.91508	-0.128	0.92424	0.98	0.90489	-0.1442	0.03097	0.2	0.78508	-0.3491	0.43075	0.86	0.92521	-0.1121	0.32324	0.59	0.91996	-0.1204	0.65702	0.78	0.91309	-0.1312	0.02105	0.09	0.85302	-0.2294	0.60368	0.81	0.90767	-0.1398	0.3097	0.61	0.92293	-0.1157
Lipidomics	PG 16_0-16_1	0.41199	0.61	0.96963	-0.0445	0.95868	0.987	0.99938	-0.0009	0.49655	0.73	0.92595	-0.111	0.03559	0.5	0.88431	-0.1774	0.6001	0.78	1.0717	0.09986	0.21398	0.36	0.93779	-0.0927	0.84798	0.91	0.99814	-0.0027	0.60133	0.81	0.97116	-0.0422	0.53619	0.74	0.96811	-0.0468
Lipidomics	PG 16_1-18_1	0.03232	0.11	0.89644	-0.1577	0.96663	0.989	1.0049	0.00703	0.20383	0.48	0.84031	-0.251	0.02966	0.5	0.82963	-0.2695	0.30467	0.58	0.89837	-0.1546	0.11218	0.23	0.90807	-0.1391	0.07151	0.17	0.85504	-0.2259	0.30304	0.57	0.93406	-0.0984	0.0636	0.44	0.85991	-0.2177
Lipidomics	PC(18:1/20:4)	0.73536	0.85	0.98221	-0.0259	0.98757	0.995	0.97904	-0.0306	0.04289	0.24	0.69154	-0.5321	0.4383	0.86	1.0946	0.13041	0.32347	0.59	0.87737	-0.1888	0.59087	0.72	1.0281	0.04	0.03716	0.11	0.81012	-0.3038	0.75123	0.88	0.96767	-0.0474	0.88198	0.96	0.99725	-0.004
Lipidomics	PE(P-16:0/22:6)	0.22049	0.41	0.92704	-0.1093	0.99906	0.999	1.0024	0.00351	0.05529	0.28	0.7716	-0.3741	0.97777	0.98	0.99568	-0.0063	0.036	0.4	0.75407	-0.4072	0.97817	0.99	1.0004	0.00063	0.0023	0.03	0.74012	-0.4342	0.60248	0.81	0.96301	-0.0544	0.23823	0.55	0.88812	-0.1712
